# Metabolic vulnerability, genetic susceptibility, and incident age-related eye diseases: a prospective cohort study

**DOI:** 10.3389/fnut.2026.1840459

**Published:** 2026-06-29

**Authors:** Yuxuan Wang, Xiaomei Wan

**Affiliations:** Department of Ophthalmology, The Affiliated Hospital of Qingdao University, Qingdao, Shandong, China

**Keywords:** age-related eye diseases, metabolic dysregulation, metabolic health, metabolic vulnerability, Polygenic risk score, risk stratification

## Abstract

**Background:**

Metabolic dysregulation is increasingly recognized as a systemic process contributing to chronic disease development, yet prospective evidence linking integrated metabolic vulnerability to age-related eye diseases remains limited. We investigated whether a biomarker-based metabolic vulnerability index (MVX) was associated with incident age-related ocular diseases and whether joint consideration of MVX and genetic susceptibility may help characterize relative risk patterns.

**Methods:**

A prospective population-based cohort of 206,311 participants from the UK Biobank was analyzed. MVX was evaluated as the primary exposure. Incident age-related macular degeneration (AMD), cataract, diabetic retinopathy (DR), and glaucoma were ascertained as outcomes. Associations were examined using Cox proportional hazards models, with hazard ratios (HRs) and 95% confidence intervals (CIs) estimated per 1–standard deviation (SD) increase in MVX. As secondary exploratory analyses, polygenic risk score (PRS) analyses were performed to explore whether metabolic vulnerability and genetic susceptibility jointly characterized relative risk patterns.

**Results:**

During follow-up, 4,144 participants developed AMD, 13,574 cataract, 1,483 DR, and 5,525 glaucoma. After multivariable adjustment for demographic, socioeconomic, clinical, and lifestyle factors, each 1-SD increase in MVX was associated with higher risks of incident AMD (HR = 1.07; 95% CI: 1.03–1.11), cataract (HR = 1.04; 95% CI: 1.02–1.06), and DR (HR = 1.11; 95% CI: 1.05–1.18), whereas no significant association was observed for glaucoma (HR = 1.00; 95% CI: 0.97–1.03). In joint analyses, individuals with both high genetic risk and elevated MVX exhibited the greatest risks of AMD (HR = 2.32; 95% CI: 2.01–2.67), cataract (HR = 1.62; 95% CI: 1.49–1.76), and DR (HR = 3.84; 95% CI: 2.91–5.06), compared with those with low genetic risk and low MVX.

**Conclusion:**

These findings suggest that MVX may be relevant to population-level patterns of risk for several age-related eye diseases. However, further studies are needed to determine whether MVX provides meaningful predictive value or clinical utility beyond conventional risk factors.

## Introduction

Age-related eye diseases, including age-related macular degeneration (AMD), cataract, diabetic retinopathy (DR), and glaucoma, are among the leading causes of visual impairment and blindness worldwide (1–3). With population aging and the increasing prevalence of metabolic disorders, the burden of these conditions is projected to rise substantially, resulting in profound consequences for vision-related quality of life and public health systems (4, 5). Although multiple clinical and demographic risk factors have been identified, current approaches to risk prediction and early identification of individuals at highest risk for developing age-related ocular diseases remain limited (6–8). In particular, most existing studies have focused on isolated metabolic traits or single biomarkers, which may fail to capture the cumulative and systemic nature of metabolic vulnerability across the life course. Consequently, there is a need for integrative markers that more comprehensively characterize metabolic risk profiles and improve understanding of population-level risk patterns for age-related eye disease.

Metabolic dysregulation has been increasingly recognized as an important contributor to the development of major ocular diseases in mid to late adulthood (9). Epidemiological evidence has linked obesity, insulin resistance, hypertension, dyslipidemia, and related lifestyle factors to elevated risks of AMD, cataract, and DR, and has suggested potential contributions to glaucomatous optic neuropathy through systemic vascular and metabolic pathways (9–11). From a biological perspective, these metabolic disturbances may affect ocular tissues through shared mechanisms, including chronic low-grade inflammation, oxidative stress, and microvascular dysfunction (12, 13). Notably, metabolic risk factors tend to cluster and progress over time rather than occur in isolation, indicating that composite measures capturing overall metabolic vulnerability may more accurately reflect real-world risk than individual biomarkers alone (14, 15).

The metabolic vulnerability index (MVX) is an integrated, biomarker-based measure designed to capture systemic metabolic vulnerability across multiple biological pathways (16). This composite approach may more accurately reflect cumulative metabolic burden under real-world conditions than individual biomarkers considered in isolation. Previous studies have shown that MVX is associated with the occurrence of diseases across multiple organ systems, including cardiovascular, neurological, and respiratory conditions, and has demonstrated utility in predicting and stratifying mortality risk in community-based populations (17–20). However, prospective evidence linking MVX to the development of age-related eye diseases remains limited, and it is unclear whether MVX provides additional population-level information beyond genetic susceptibility.

Genetic susceptibility represents an important background determinant of age-related eye diseases (21, 22). Whereas Polygenic risk scores (PRS) summarize relatively stable inherited predisposition, MVX reflects systemic metabolic vulnerability that is more dynamic and potentially modifiable (16, 23). We therefore considered it informative, as a secondary objective, to explore whether these two dimensions might jointly characterize relative risk patterns for age-related eye diseases at the population level.

Accordingly, we conducted a prospective cohort study in the UK Biobank to examine whether MVX was associated with the incidence of AMD, cataract, DR, and glaucoma. Our primary objective was to evaluate the prospective association between MVX and these outcomes using Cox proportional hazards models, with MVX modeled primarily as a continuous exposure per 1–standard deviation (SD) increase. As secondary analyses, we further characterized these associations using alternative exposure parameterizations and restricted cubic splines, and explored whether metabolic vulnerability and genetic susceptibility jointly characterized relative risk patterns.

## Methods

### Study population

**Figure 1:**
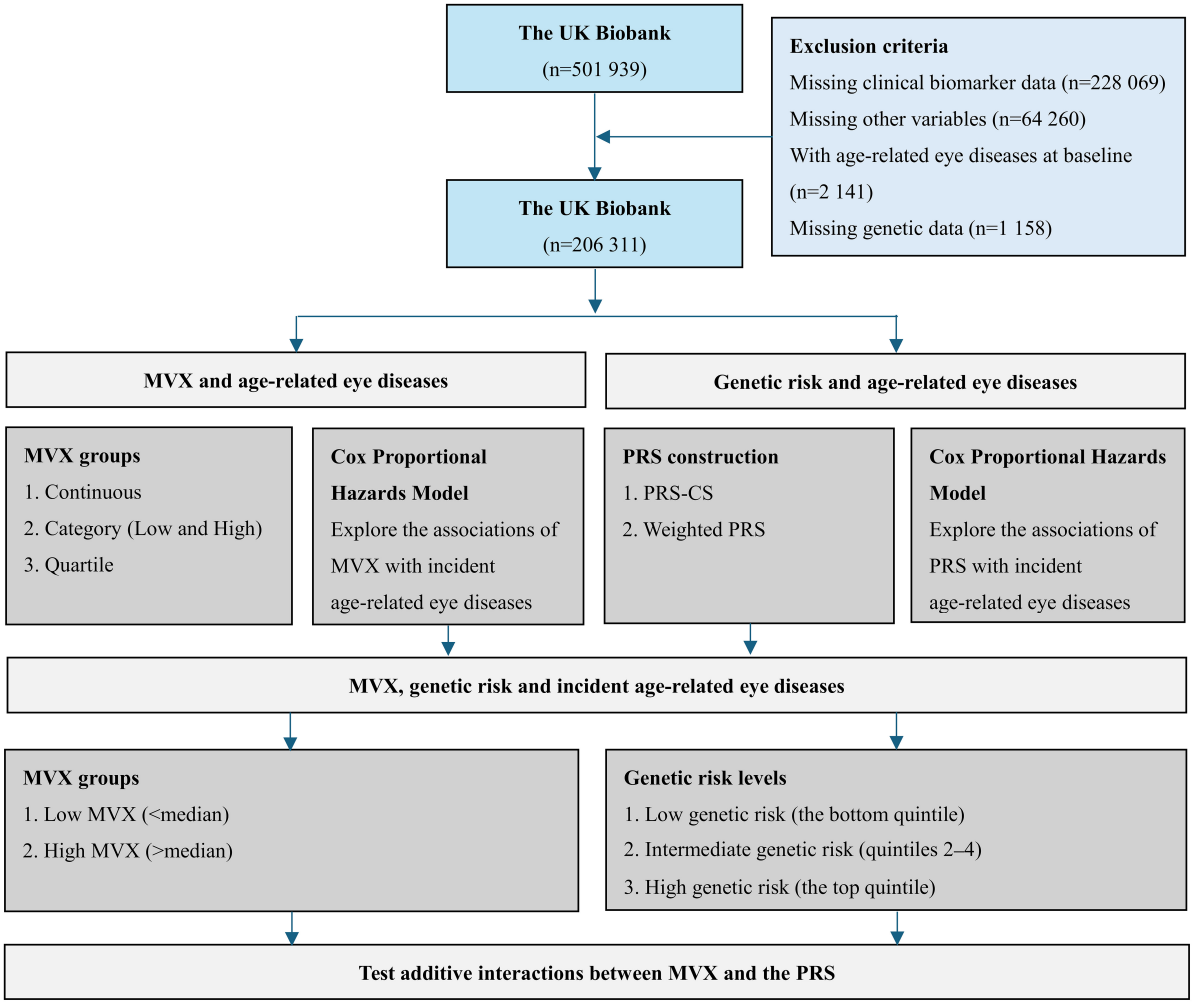
Overview of the study design and analytical process. MVX, metabolic vulnerability index; PRS, polygenic risk score; CS, continuous shrinkage.

The UK Biobank is a large prospective cohort study that recruited over 500,000 participants aged 40–69 years across the United Kingdom between 2006 and 2010. Baseline assessments comprised detailed questionnaires, physical measurements, and the collection of biological samples, with ongoing follow-up through linkage to health-related records (24). Of the full cohort (*n* = 501,939), participants were excluded if they had: (1) missing biomarker data required for MVX calculation (*n* = 228,069); (2) missing covariate data (*n* = 64,260); (3) prevalent age-related eye diseases at baseline (*n* = 2,141); or (4) missing genetic data (*n* = 1,158). After applying these exclusion criteria, 206,311 participants were included in the primary analyses (Figure 1). The UK Biobank study received ethical approval from the North West Multicentre Research Ethics Committee.

### Assessment of metabolic vulnerability

MVX was calculated according to a previously published, sex-specific framework based on six circulating biomarkers quantified by nuclear magnetic resonance (NMR) spectroscopy, including glycoprotein acetyls (GlycA), small HDL particle (sHDL), leucine, valine, isoleucine, and citrate. Briefly, the inflammation vulnerability index (IVX) was derived from GlycA, sHDL, and their interaction term, whereas the metabolic malnutrition index (MMX) was derived from leucine, valine, isoleucine, and citrate, including quadratic terms where applicable. MVX was subsequently calculated as a function of IVX, ln (MMX), and their interaction using sex-specific coefficients derived from the original published framework. Prior to score construction, each biomarker was winsorized at the 1st and 99th percentiles to reduce the influence of extreme values, and the resulting IVX, MMX, and MVX scores were normalized to a range of 1–100. Full sex-specific equations, biomarker definitions, and score-construction procedures are provided in the Supplementary Methods. For the primary analyses, MVX was analyzed as a continuous variable and standardized per 1–SD increase. IVX and MMX were evaluated in secondary analyses using continuous and categorical parameterizations. For categorical analyses, each index was further classified into low and high groups according to the median value (low group as the reference) and into quartiles (Q1–Q4, with Q1 as the reference).

### Construction of PRS

To quantify individual-level genetic susceptibility to age-related eye diseases, disease-specific polygenic risk scores (PRS) for AMD, cataract, DR, and glaucoma were constructed using two approaches: (1) a curated weighted PRS based on established risk variants and (2) a genome-wide PRS generated using PRS-CS (continuous shrinkage). For the weighted PRS, lead single-nucleotide polymorphisms (SNPs) associated with AMD, cataract, DR, and glaucoma were extracted from previously published studies curated in the PGS Catalog, comprising 19, 647, 39, and 243 SNPs, respectively (25–27) (Tables S1–S4). SNPs were aligned to the reported effect allele, and each variant was weighted by the corresponding log-odds ratio. The weighted PRS was calculated as the sum of effect-allele dosages multiplied by their respective weights across all included SNPs. Genome-wide PRS were constructed using PRS-CS, which applies Bayesian regression with continuous shrinkage priors. This approach incorporates linkage disequilibrium (LD) information from an external European reference panel derived from the 1,000 Genomes Project to estimate posterior SNP effect sizes without reliance on arbitrary *P*-value thresholds (28). Genome-wide PRS were calculated by summing SNP dosages weighted by PRS-CS posterior effect estimates based on external genome-wide association study (GWAS) summary statistics for each outcome (29).

For interaction analyses, genetic risk based on each PRS was categorized into low (quintile 1), intermediate (quintiles 2–4), and high (quintile 5) risk groups, consistent with prior literature (30). All PRS values were standardized prior to inclusion in regression models.

### Assessment of age-related eye diseases

Four common eye diseases with substantial age-related burden were investigated: AMD, cataract, DR, and glaucoma. The primary outcomes were incident age-related eye diseases. Incident cases were ascertained through linked hospital inpatient records from the UK Biobank Hospital Episode Statistics (HES) database using International Classification of Diseases codes (ICD-10 and ICD-9) (Table S5). For each outcome, incident disease was defined as the first recorded diagnosis occurring after the baseline assessment date. To restrict analyses to incident events, participants with a recorded diagnosis of the corresponding eye disease on or before baseline were excluded. For each outcome, the event date was defined as the date of the first qualifying diagnosis recorded in the HES data.

### Covariates

Covariates were selected *a priori* based on previous literature and their potential associations with both MVX and age-related eye diseases. Baseline variables included chronological age (years), sex (male or female), ethnicity (white, mixed, Asian, black, or others), education level (degree-level or professional qualification vs. other levels), body mass index (BMI, kg/m^2^), Townsend Deprivation Index, physical activity (high, moderate, or low), sleep duration (long ≥8 h, moderate 7–8 h, or short ≤ 6 h), healthy diet (yes or no), smoking status (yes or no), and alcohol consumption (yes or no). Physical activity was categorized as high (≥3,000 MET-min/week), moderate (600–3,000 MET-min/week), or low (< 600 MET-min/week) according to total weekly metabolic equivalent task minutes (31). Definitions of smoking status, alcohol consumption, and healthy diet were based on previously published criteria; detailed variable definitions are provided in the Supplementary material (32, 33) (Table S5).

### Statistical analyses

Baseline characteristics were summarized as means ± SD for continuous variables and counts (%) for categorical variables. The primary analyses evaluated the associations between continuous MVX and incident age-related eye diseases using Cox proportional hazards models, with HRs and 95% CIs estimated per 1-SD increase in MVX. As secondary analyses, MVX was additionally examined using categorical parameterizations, including median-based groups (low vs. high, with low as the reference) and quartiles (Q1–Q4, with Q1 as the reference), to assess the robustness of the findings across alternative exposure specifications. IVX and MMX were also evaluated in secondary analyses using continuous and categorical parameterizations. Restricted cubic spline functions were applied as secondary analyses to characterize potential non-linearity in the associations between MVX and each outcome, using four knots placed at the 5th, 35th, 65th, and 95th percentiles of MVX, with the median MVX value serving as the reference.

Two multivariable models were fitted. Model 1 was adjusted for age, sex, ethnicity, education level, BMI, and the Townsend Deprivation Index. Model 2 was further adjusted for physical activity, sleep duration, smoking status, alcohol consumption, and healthy diet. The proportional hazards assumption was assessed using Schoenfeld residuals.

Genetic susceptibility was evaluated using standardized PRS. Associations between PRS and each outcome were assessed using Cox models adjusted for the covariates described above. As secondary exploratory analyses, we further explored whether metabolic vulnerability and genetic susceptibility jointly characterized relative risk patterns through joint classification of MVX (low vs. high) and PRS categories (low: quintile 1; intermediate: quintiles 2–4; high: quintile 5). Additive interaction between MVX and PRS was quantified using the relative excess risk due to interaction (RERI) and the attributable proportion due to interaction (AP), with 95% CIs estimated using 1,000 bootstrap resamples (36). Subgroup analyses were also conducted as exploratory analyses, stratified by sex, age group, BMI category, smoking status, and physical activity, to explore potential heterogeneity of associations.

All statistical tests were two-sided, and *P* < 0.05 was considered statistically significant. All analyses were conducted within the UK Biobank Research Analysis Platform.

## Results

### Participants

Baseline characteristics of the included participants are presented in Table 1. The mean chronological age at baseline was 56.19 ± 8.10 years, and 99,676 participants (48.31%) were male. The majority of participants were of White ethnicity (95.4%), and 35.1% reported a degree-level or professional qualification. The mean BMI was 27.29 ± 4.67 kg/m^2^, and the mean Townsend Deprivation Index was −1.49 ± 3.00. During follow-up, incident cases included 4,144 AMD (2.0%), 13,574 cataract (6.6%), 1,483 DR (0.7%), and 5,525 glaucoma (2.7%). Distributions of MVX, IVX, and MMX stratified by age-related eye disease status are shown in Supplementary Figures S1–S3.

**Table 1:** Baseline characteristics among participants.

Variables	Total	Male	Female
Chronological age (years)	56.19 ± 8.10	56.55 ± 8.19	55.87 ± 8.00
Ethnicity			
White	196,744 (95.4%)	95,061 (95.4%)	101,683 (95.4%)
Mixed	1,125 (0.5%)	448 (0.4%)	677 (0.6%)
Asian	3,141 (1.5%)	1,784 (1.8%)	1,357 (1.3%)
Black	2,577 (1.2%)	1,088 (1.1%)	1,489 (1.4%)
Others	2,724 (1.3%)	1,297 (1.3%)	1,427 (1.3%)
Education level			
Degree level or professional education	72,455 (35.1%)	35,907 (36.0%)	36,548 (34.3%)
Other levels	133,856 (64.9%)	63,771 (64.0%)	70,085 (65.7%)
BMI (kg/m^2^)	27.29 ± 4.67	27.75 ± 4.16	26.86 ± 5.06
Townsend deprivation index	−1.49 ± 3.00	−1.49 ± 3.05	−1.49 ± 2.95
Physical activity			
Low	37,845 (18.3%)	18,532 (18.6%)	19,313 (18.1%)
Moderate	103,926 (50.4%)	48,575 (48.7%)	55,351 (51.9%)
High	64,540 (31.3%)	32,571 (32.7%)	31,969 (30.0%)
Sleep duration			
Short	52,399 (25.4%)	25,800 (25.9%)	26,599 (24.9%)
Moderate	141,572 (68.6%)	68,260 (68.5%)	73,312 (68.8%)
Long	12,340 (6.0%)	5,618 (5.6%)	6,722 (6.3%)
Healthy diet			
No	175,581 (85.1%)	87,402 (87.7%)	88,179 (82.7%)
Yes	30,730 (14.9%)	12,276 (12.3%)	18,454 (17.3%)
Smoking			
No	112,322 (54.4%)	48,121 (48.3%)	64,201 (60.2%)
Yes	93,989 (45.6%)	51,557 (51.7%)	42,432 (39.8%)
Alcohol			
No	110,336 (53.5%)	56,238 (56.4%)	54,098 (50.7%)
Yes	95,975 (46.5%)	43,440 (43.6%)	52,535 (49.3%)
AMD			
No	202,167 (98.0%)	97,923 (98.2%)	104,244 (97.8%)
Yes	4,144 (2.0%)	1,755 (1.8%)	2,389 (2.2%)
DR			
No	204,828 (99.3%)	98,716 (99.0%)	106,112 (99.5%)
Yes	1,483 (0.7%)	962 (1.0%)	521 (0.5%)
Cataract			
No	192,737 (93.4%)	93,791 (94.1%)	98,946 (92.8%)
Yes	13,574 (6.6%)	5,887 (5.9%)	7,687 (7.2%)
Glaucoma			
No	200,786 (97.3%)	96,890 (97.2%)	103,896 (97.4%)
Yes	5,525 (2.7%)	2,788 (2.8%)	2,737 (2.6%)
Citrate (μmol/L)	65.14 ± 12.65	63.54 ± 12.21	66.63 ± 12.86
Isoleucine (μmol/L)	51.21 ± 17.48	54.80 ± 17.27	47.86 ± 17.01
Glycoprotein acetyls (μmol/L)	806.71 ± 116.23	807.69 ± 114.54	805.81 ± 117.77
Leucine (μmol/L)	104.44 ± 27.88	112.55 ± 27.11	96.86 ± 26.42
Small HDL particle (μmol/L)	9.77 ± 1.29	9.69 ± 1.26	9.84 ± 1.31
Valine (μmol/L)	210.65 ± 42.21	220.93 ± 40.94	201.04 ± 41.10
IVX	39.40 ± 18.23	28.15 ± 14.10	49.91 ± 15.12
MMX	46.42 ± 15.70	53.05 ± 7.58	40.22 ± 18.54
MVX	40.38 ± 15.06	32.36 ± 13.04	47.88 ± 12.77

Values are presented as mean ± SD or n (%). AMD, age-related macular degeneration; DR, diabetic retinopathy; IVX, inflammation vulnerability index; MMX, metabolic malnutrition index; MVX, metabolic vulnerability index.

### MVX and incident age-related eye diseases risk

In the primary analyses, higher continuous MVX was associated with increased risks of incident AMD, cataract, and DR. Per 1-SD increase in MVX, the HRs were 1.07 (95% CI: 1.03–1.11) for AMD, 1.05 (95% CI: 1.03–1.07) for cataract, and 1.12 (95% CI: 1.06–1.19) for DR after adjustment for age, sex, ethnicity, education level, BMI, and the Townsend Deprivation Index. No association was observed between MVX and glaucoma (HR = 1.00; 95% CI: 0.97–1.03). These associations were materially unchanged after further adjustment for physical activity, sleep duration, smoking status, alcohol consumption, and healthy diet.

**Figure 2:**
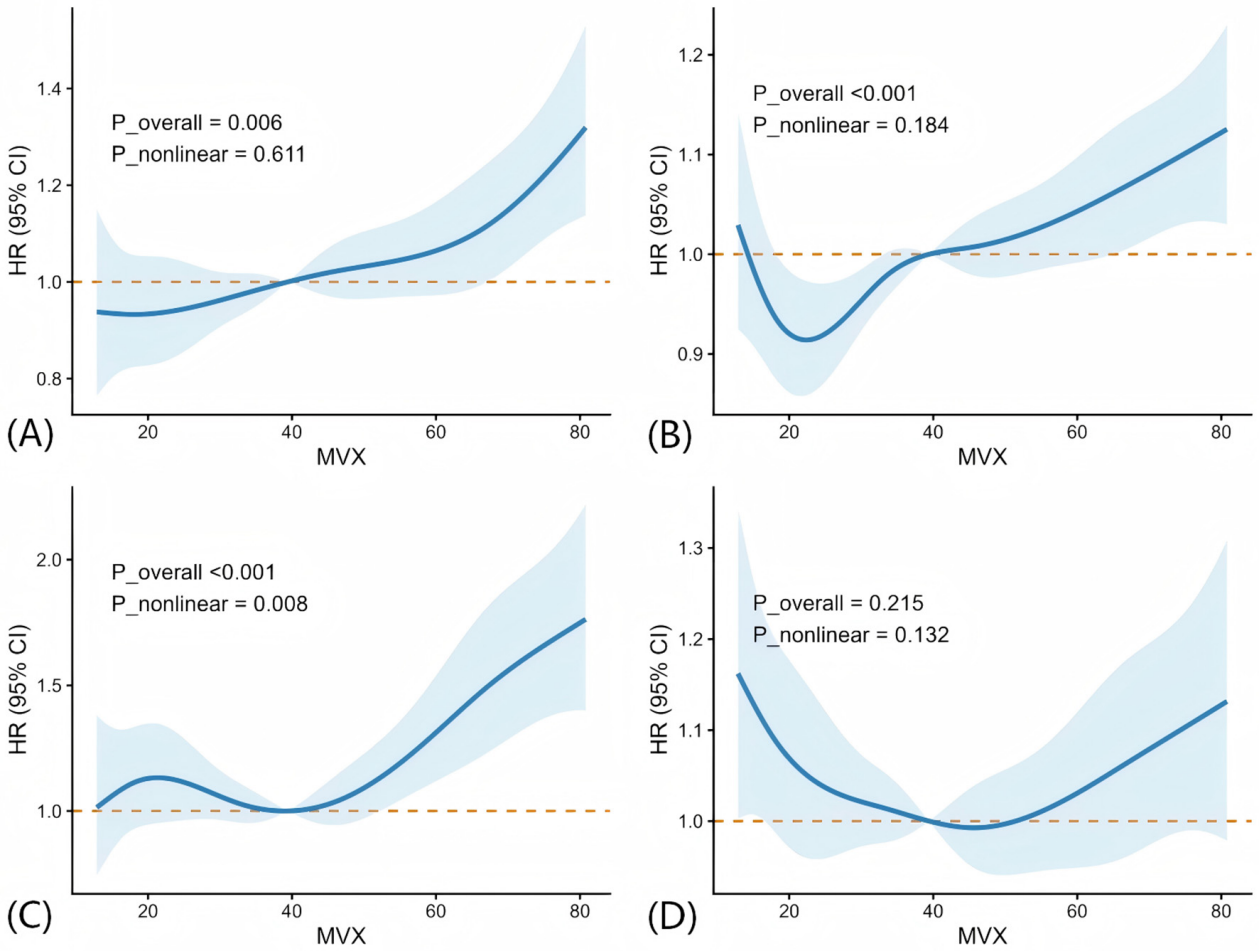
Linear and non-linear association between MVX and incident age-related eye diseases. Chronological age, sex, ethnicity, education, BMI, and Townsend deprivation index, physical activity, sleep duration, smoking, alcohol consumption, and healthy diet were adjusted in the RCS analyses. **(A)** MVX and age-related macular degeneration; **(B)** MVX and cataract; **(C)** MVX and diabetic retinopathy; **(D)** MVX and glaucoma. MVX: metabolic vulnerability index.

**Figure 3:**
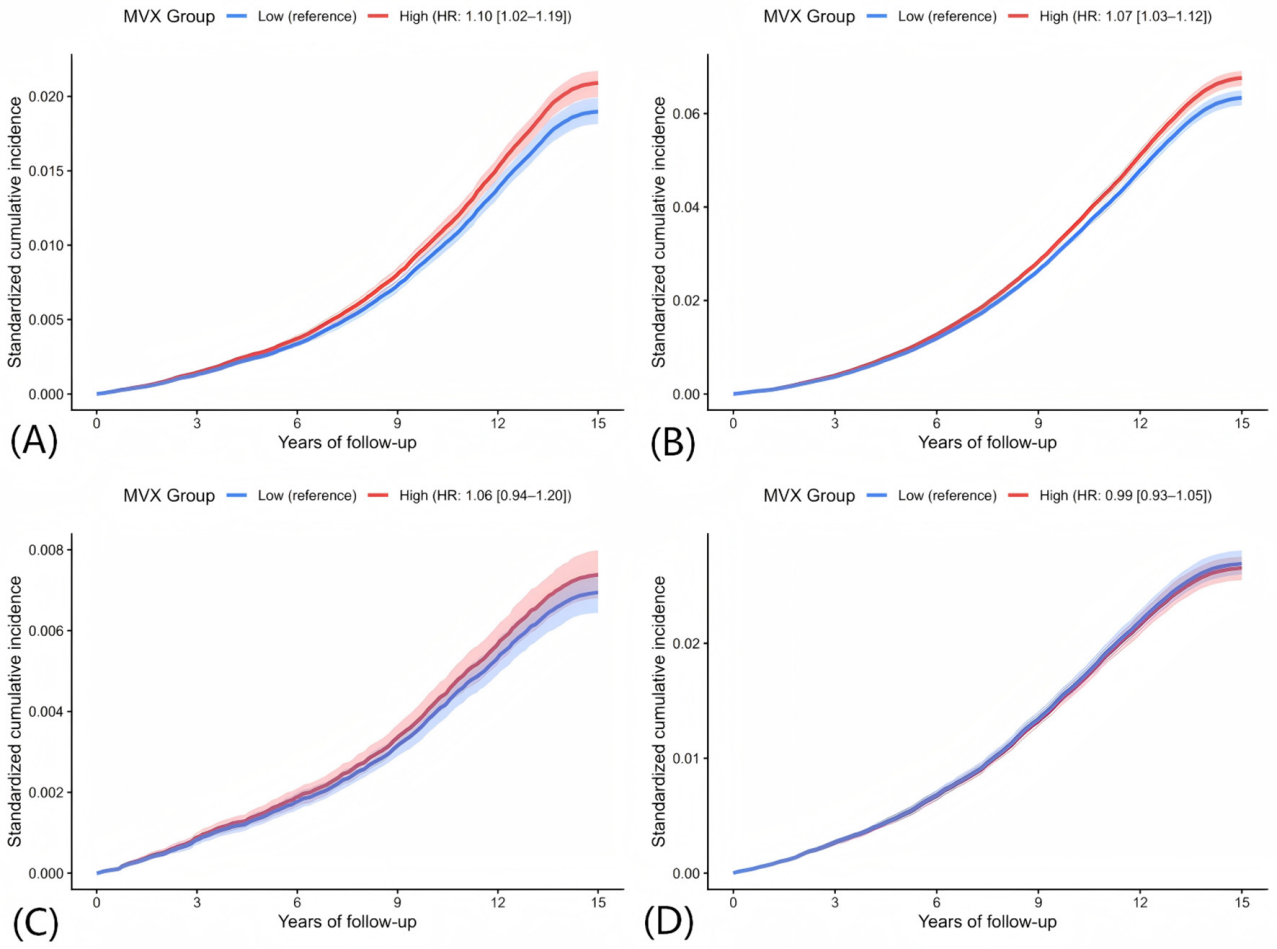
Associations between MVX and incident age-related eye diseases risk among different MVX groups. Chronological age, sex, ethnicity, education, BMI, and Townsend deprivation index, physical activity, sleep duration, smoking, alcohol consumption, and healthy diet were adjusted in the analyses. **(A)**, MVX and age-related macular degeneration; **(B)**, MVX and cataract; **(C)**, MVX and diabetic retinopathy; **(D)**, MVX and glaucoma. MVX, metabolic vulnerability index.

As secondary analyses, restricted cubic spline analyses and categorical parameterizations were used to further characterize the shape and robustness of the observed associations. The MVX–risk relationships for AMD and cataract showed broadly monotonic increases without evidence of non-linearity (*P*_nonlinear > 0.05), whereas a non-linear association was observed for DR (*P*_nonlinear = 0.008), with risk increasing more steeply at higher MVX levels. No significant association between MVX and glaucoma was observed in spline analyses (*P*_overall = 0.215) (Figure 2). When MVX was dichotomized at the median value (39.42), participants in the high MVX group had higher risks of AMD (HR = 1.10; 95% CI: 1.02–1.19) and cataract (HR = 1.07; 95% CI: 1.03–1.12) compared with those in the low MVX group, whereas no significant associations were observed for DR or glaucoma (Figure 3). In quartile analyses, associations generally strengthened across increasing MVX categories. Compared with Q1, participants in Q4 had higher risks of AMD (HR = 1.20; 95% CI: 1.08–1.33), cataract (HR = 1.12; 95% CI: 1.06–1.19), and DR (HR = 1.22; 95% CI: 1.03–1.44) (Table 2).

**Table 2:** Association between MVX and incident age-related eye diseases risk.

Exposure	Outcome	Events/Total	Model 1		Model 2	
			HR (95% CI)	*P*-value	HR (95% CI)	*P*-value
MVX (continuous)	AMD					
Per 1-SD		4,144/206,311	1.07 (1.04–1.11)	**< 0.001**	1.07 (1.03–1.11)	**< 0.001**
MVX (category)	AMD					
Low (< 39.42)		1778/103,156	1.00 (reference)	–	1.00 (reference)	–
High (≥39.42)		2366/103,155	1.11 (1.03–1.19)	**0.008**	1.10 (1.02–1.19)	**0.009**
MVX (quartile)	AMD					
Q1 (< 28.95)		840/51,578	1.00 (reference)	–	1.00 (reference)	–
Q2 (28.95–39.42)		938/51,578	1.07 (0.97–1.18)	0.177	1.06 (0.96–1.17)	0.226
Q3 (39.42–50.28)		1,058/51,578	1.11 (1.00–1.23)	0.049	1.10 (0.99–1.22)	0.063
Q4 (>50.28)		1,308/51,577	1.20 (1.08–1.34)	**< 0.001**	1.20 (1.08–1.33)	**< 0.001**
MVX (continuous)	Cataract					
Per 1-SD		13,574/206,311	1.05 (1.03–1.07)	**< 0.001**	1.04 (1.02–1.06)	**< 0.001**
MVX (category)	Cataract					
Low (< 39.42)		5,967/103,156	1.00 (reference)	–	1.00 (reference)	–
High (≥39.42)		7,607/103,155	1.08 (1.03–1.12)	**< 0.001**	1.07 (1.03–1.12)	**< 0.001**
MVX (quartile)	Cataract					
Q1 (< 28.95)		2,850/51,578	1.00 (reference)	–	1.00 (reference)	–
Q2 (28.95–39.42)		3,117/51,578	1.05 (1.00–1.11)	0.076	1.04 (0.99–1.10)	0.107
Q3 (39.42–50.28)		3,486/51,578	1.09 (1.03–1.15)	**0.003**	1.08 (1.02–1.15)	**0.006**
Q4 (>50.28)		4,121/51,577	1.13 (1.07–1.20)	**< 0.001**	1.12 (1.06–1.19)	**< 0.001**
MVX (continuous)	DR					
Per 1-SD		1,483/206,311	1.12 (1.06–1.19)	**< 0.001**	1.11 (1.05–1.18)	**< 0.001**
MVX (category)	DR					
Low (< 39.42)		772/103,156	1.00 (reference)	–	1.00 (reference)	–
High (≥39.42)		711/103,155	1.08 (0.95–1.21)	0.240	1.06 (0.94–1.20)	0.324
MVX (quartile)	DR					
Q1 (< 28.95)		416/51,578	1.00 (reference)	–	1.00 (reference)	–
Q2 (28.95–39.42)		356/51,578	0.94 (0.82–1.09)	0.435	0.95 (0.82–1.09)	0.451
Q3 (39.42–50.28)		280/51,578	0.89 (0.76–1.05)	0.176	0.90 (0.76–1.06)	0.195
Q4 (>50.28)		431/51,577	1.24 (1.05–1.47)	**0.009**	1.22 (1.03–1.44)	**0.022**
MVX (continuous)	Glaucoma					
Per 1-SD		5,525/206,311	1.00 (0.97–1.04)	0.865	1.00 (0.97–1.03)	0.981
MVX (category)	Glaucoma					
Low (< 39.42)		2768/103,156	1.00 (reference)	–	1.00 (reference)	–
High (≥39.42)		2757/103,155	0.99 (0.93–1.05)	0.728	0.99 (0.93–1.05)	0.657
MVX (quartile)	Glaucoma					
Q1 (< 28.95)		1,447/51,578	1.00 (reference)	–	1.00 (reference)	–
Q2 (28.95–39.42)		1,321/51,578	0.94 (0.87–1.02)	0.131	0.94 (0.87–1.02)	0.132
Q3 (39.42–50.28)		1,313/51,578	0.94 (0.86–1.02)	0.155	0.94 (0.86–1.02)	0.146
Q4 (>50.28)		1,444/51,577	0.97 (0.89–1.06)	0.522	0.97 (0.88–1.06)	0.453

Model 1 adjusted for chronological age, sex, ethnicity, education, BMI, and Townsend deprivation index. Model 2 further adjusted for physical activity, sleep duration, smoking, alcohol consumption, and healthy diet. Bold values indicate statistical significance at *P* < 0.05. MVX, metabolic vulnerability index; AMD, age-related macular degeneration; DR: diabetic retinopathy.

### Subgroup analyses

**Figure 4:**
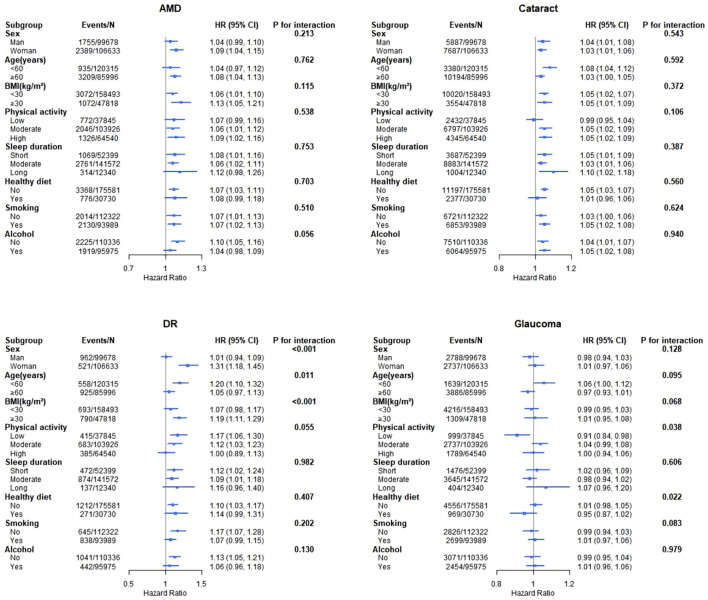
Associations between MVX and incident age-related eye diseases across subgroups. Chronological age, sex, ethnicity, education, BMI, and Townsend deprivation index, physical activity, sleep duration, smoking, alcohol consumption, and healthy diet were adjusted in the subgroup analyses. BMI, body mass index; AMD, age-related macular degeneration; DR, diabetic retinopathy.

As secondary exploratory analyses, subgroup analyses were conducted to evaluate whether the associations between MVX and incident age-related eye diseases differed across participant subgroups (Figure 4). For AMD and cataract, the associations were generally consistent across subgroups, with no evidence of effect modification by sex, age, BMI, physical activity, sleep duration, healthy diet, smoking status, or alcohol consumption (all *P* for interaction > 0.05). For DR, significant effect modification was observed by sex (*P* for interaction < 0.001), age group (*P* for interaction = 0.011), and BMI category (*P* for interaction < 0.001). The association between MVX and incident DR was stronger among women than men, stronger among participants aged < 60 years than those aged ≥60 years, and stronger among participants with BMI ≥30 kg/m^2^ than those with BMI < 30 kg/m^2^. For glaucoma, no statistically significant overall association with MVX was observed in the main analyses; however, heterogeneity across subgroups was suggested for physical activity (*P* for interaction = 0.038) and healthy diet (*P* for interaction = 0.022).

### PRS and incident age-related eye diseases risk

As secondary exploratory analyses, we further examined whether genetic susceptibility, quantified using PRS, was associated with incident age-related eye diseases. Higher PRS were generally associated with increased risks of incident age-related eye diseases after adjustment for all covariates (Table 3 and Tables S6–S9). Using PRS-CS, each 1-SD increase in PRS was associated with higher risks of incident AMD (HR = 1.36; 95% CI: 1.32–1.40), DR (HR = 1.55; 95% CI: 1.47–1.63), and glaucoma (HR = 1.37; 95% CI: 1.34–1.41), whereas no significant association was observed for cataract (HR = 1.01; 95% CI: 0.99–1.02). When categorized into genetic risk groups, participants in the highest PRS category exhibited the greatest risks of AMD (HR = 2.16; 95% CI: 1.96–2.38), DR (HR = 3.51; 95% CI: 2.90–4.25), and glaucoma (HR = 2.36; 95% CI: 2.16–2.58), compared with those in the lowest risk group (Figure S4). For the weighted PRS, each 1-SD increase was associated with higher risks of incident AMD (HR = 1.36; 95% CI: 1.32–1.40), cataract (HR = 1.16; 95% CI: 1.14–1.18), DR (HR = 1.17; 95% CI: 1.12–1.23), and glaucoma (HR = 1.27; 95% CI: 1.24–1.31) (Figure S5). Consistently, participants in the highest weighted PRS category had the greatest risks of age-related eye diseases compared with those in the lowest category.

**Table 3:** Association between PRS and age-related eye diseases risk.

Exposures	Outcomes	Model 1		Model 2	
		HR (95% CI)	*P*-value	HR (95% CI)	*P*-value
PRS-CS	AMD				
Per 1 SD increase		1.36 (1.32–1.40)	**< 0.001**	1.36 (1.32–1.40)	**< 0.001**
Low risk		1.00 (reference)	**–**	1.00 (reference)	**–**
Intermediate risk		1.27 (1.16–1.38)	**< 0.001**	1.26 (1.16–1.38)	**< 0.001**
High genetic risk		2.16 (1.96–2.38)	**< 0.001**	2.16 (1.96–2.38)	**< 0.001**
Weighted PRS	Cataract				
Per 1 SD increase		1.16 (1.14–1.18)	**< 0.001**	1.16 (1.14–1.18)	**< 0.001**
Low risk		1.00 (reference)	**–**	1.00 (reference)	**–**
Intermediate risk		1.24 (1.18–1.29)	**< 0.001**	1.24 (1.18–1.29)	**< 0.001**
High genetic risk		1.52 (1.44–1.61)	**< 0.001**	1.52 (1.44–1.61)	**< 0.001**
PRS-CS	DR				
Per 1 SD increase		1.56 (1.48–1.64)	**< 0.001**	1.55 (1.47–1.63)	**< 0.001**
Low risk		1.00 (reference)	**–**	1.00 (reference)	**–**
Intermediate risk		1.90 (1.58–2.29)	**< 0.001**	1.89 (1.57–2.27)	**< 0.001**
High genetic risk		3.57 (2.95–4.33)	**< 0.001**	3.51 (2.90–4.25)	**< 0.001**
PRS-CS	Glaucoma				
Per 1 SD increase		1.37 (1.34–1.41)	**< 0.001**	1.37 (1.34–1.41)	**< 0.001**
Low risk		1.00 (reference)	**–**	1.00 (reference)	**–**
Intermediate risk		1.53 (1.41–1.66)	**< 0.001**	1.53 (1.41–1.66)	**< 0.001**
High genetic risk		2.36 (2.16–2.58)	**< 0.001**	2.36 (2.16–2.58)	**< 0.001**

Model 1 adjusted for chronological age, sex, ethnicity, education, BMI, and Townsend deprivation index. Model 2 further adjusted for physical activity, sleep duration, smoking, alcohol consumption, and healthy diet. Bold values indicate statistical significance at *P* < 0.05. PRS, polygenic risk score; CS, continuous shrinkage. BMI, body mass index; AMD, age-related macular degeneration; DR: diabetic retinopathy.

To support the subsequent exploratory joint analyses, the predictive performance of PRS-CS and the weighted PRS was compared using Harrell's C-index. PRS-CS demonstrated slightly better discrimination for AMD (0.7570 vs. 0.7567), DR (0.8000 vs. 0.7850), and glaucoma (0.7128 vs. 0.7056), whereas the weighted PRS showed superior performance for cataract (0.7391 vs. 0.7355). Accordingly, PRS-CS was selected as the primary PRS for AMD, DR, and glaucoma, and the weighted PRS was selected for cataract in the subsequent joint analyses.

### Joint effects and interactions of MVX and genetic risk

As secondary exploratory analyses, we further examined whether MVX and genetic susceptibility jointly characterized relative risk patterns for age-related eye diseases. Using the primary PRS selected based on C-index performance (PRS-CS for AMD, DR, and glaucoma; weighted PRS for cataract), joint analyses indicated that genetic susceptibility was strongly associated with incident age-related eye diseases, and participants with concomitantly high genetic risk and high MVX generally exhibited the greatest risks (Table 4). Compared with the low genetic risk/low MVX group, the high genetic risk/high MVX group had the highest risks of incident AMD (HR = 2.32; 95% CI: 2.01–2.67), cataract (HR = 1.62; 95% CI: 1.49–1.76), and DR (HR = 3.84; 95% CI: 2.91–5.06). For glaucoma, genetic risk remained strongly associated with disease incidence, whereas MVX did not meaningfully further increase risk within genetic strata. Specifically, among participants with high genetic risk, the HRs for glaucoma were comparable between those with high MVX (HR = 2.28; 95% CI: 2.00–2.60) and those with low MVX (HR = 2.48; 95% CI: 2.19–2.80).

**Table 4:** Joint effects of MVX and genetic risk on age-related eye diseases risk.

Subgroup	Events/Total	Incidence per 100,000 PY	HR (95% CI)	*P*-value
**AMD**				
Low genetic risk				
Low MVX	281/20,876	83.13	1.00 (reference)	–
High MVX	325/20,387	98.45	0.98 (0.83–1.16)	0.824
Intermediate genetic risk				
Low MVX	985/61,852	98.44	1.20 (1.06–1.38)	**0.006**
High MVX	1,279/61,934	127.82	1.29 (1.13–1.48)	**< 0.001**
High genetic risk				
Low MVX	512/20,428	155.52	1.91 (1.65–2.21)	**< 0.001**
High MVX	762/20,834	227.99	2.32 (2.01–2.67)	**< 0.001**
**Cataract**				
Low genetic risk				
Low MVX	989/20,606	300.48	1.00 (reference)	–
High MVX	1,231/20,657	374.46	1.04 (0.95–1.13)	0.412
Intermediate genetic risk				
Low MVX	3,557/61,968	361.28	1.21 (1.13–1.30)	**< 0.001**
High MVX	4,523/61,818	463.38	1.30 (1.21–1.40)	**< 0.001**
High genetic risk				
Low MVX	1,421/20,582	437.84	1.48 (1.36–1.60)	**< 0.001**
High MVX	1,853/20,680	573.34	1.62 (1.49–1.76)	**< 0.001**
**DR**				
Low genetic risk				
Low MVX	68/20,644	20.25	1.00 (reference)	–
High MVX	62/20,619	18.46	1.05 (0.74–1.49)	0.774
Intermediate genetic risk				
Low MVX	425/61,991	42.22	1.90 (1.47–2.45)	**< 0.001**
High MVX	385/61,795	38.34	1.98 (1.52–2.59)	**< 0.001**
High genetic risk				
Low MVX	279/20,521	84.07	3.40 (2.60–4.43)	**< 0.001**
High MVX	264/20,741	78.59	3.84 (2.91–5.06)	**< 0.001**
**Glaucoma**				
Low genetic risk				
Low MVX	349/20,843	103.58	1.00 (reference)	–
High MVX	354/20,420	107.21	1.01 (0.87–1.18)	0.85
Intermediate genetic risk				
Low MVX	1,573/61,723	158.38	1.53 (1.36–1.72)	**< 0.001**
High MVX	1,611/62,063	161.26	1.55 (1.37–1.75)	**< 0.001**
High genetic risk				
Low MVX	846/20,590	257.56	2.48 (2.19–2.80)	**< 0.001**
High MVX	792/20,672	239.56	2.28 (2.00–2.60)	**< 0.001**

Chronological age, sex, ethnicity, education, BMI, and Townsend deprivation index, physical activity, sleep duration, smoking, alcohol consumption, and healthy diet were adjusted in the analyses. Bold values indicate statistical significance at *P* < 0.05. BMI, body mass index; AMD, age-related macular degeneration; DR, diabetic retinopathy.

Additive interaction analyses provided evidence of synergism between MVX and genetic risk for AMD only. Among participants with high PRS, high MVX was associated with a positive additive interaction on the risk of incident AMD, as indicated by a relative excess risk due to interaction (RERI) of 0.42 (95% CI: 0.15–0.70) and an attributable proportion (AP) of 0.18 (95% CI: 0.07–0.30). No evidence of additive interaction was observed for cataract, DR, or glaucoma (Table S10).

## Discussion

In this prospective cohort study, the primary finding was that higher MVX was associated with increased risks of incident AMD, cataract, and DR, but not glaucoma, after multivariable adjustment. Secondary analyses using alternative exposure parameterizations and restricted cubic splines were broadly consistent with these findings and further suggested a non-linear association between MVX and DR, with risk increasing more prominently at higher MVX levels. Additional exploratory analyses further suggested that metabolic vulnerability and genetic susceptibility may jointly characterize relative risk patterns for age-related eye diseases, particularly for AMD. Taken together, these findings support the relevance of MVX as a biomarker of metabolic vulnerability associated with several age-related eye diseases at the population level, although further work is required to determine whether it offers meaningful predictive or clinical utility beyond conventional risk factors.

Previous studies have linked adverse cardiometabolic profiles, including obesity, dysglycaemia or diabetes, hypertension, and dyslipidaemia, to increased risks of AMD, cataract, and DR, although the strength and consistency of these associations have varied across populations and study designs (9, 34–37). However, most prior work has focused on individual metabolic traits or composite clinical definitions, and prospective evidence capturing the joint burden of biomarker-based metabolic vulnerability across multiple ocular endpoints remains limited. The present findings indicate that an integrated biomarker-based index of metabolic vulnerability, as reflected by MVX, is prospectively associated with incident AMD, cataract, and DR, whereas no association was observed for glaucoma. The observed non-linear pattern for DR suggests that the relationship between metabolic vulnerability and DR risk may differ across the MVX distribution, with a more pronounced increase in risk at higher MVX levels than would be expected under a linear assumption. In addition, a growing body of PRS research has demonstrated genetic risk stratification for age-related eye diseases (38–40). By comparing two PRS construction strategies and carrying forward the better-performing score for each outcome, the present study suggests that metabolic vulnerability and genetic susceptibility may provide complementary population-level information, although the incremental predictive value of such integration remains to be established.

The observed associations between higher MVX and incident AMD, cataract, and DR are biologically plausible, as MVX captures integrated metabolic vulnerability across multiple pathways that converge on ocular aging (17). Metabolic dysregulation is closely linked to chronic low-grade inflammation, oxidative stress, endothelial dysfunction, and microvascular impairment, which may influence the choroid, retina, and other ocular tissues through impaired perfusion, altered lipid handling, and cumulative cellular injury (41, 42). These processes have been implicated in retinal pigment epithelium dysfunction and drusen-related changes in AMD, lens protein oxidation and glycation in cataract, and capillary damage with breakdown of the blood–retinal barrier in DR (43–45). The non-linear association observed for DR may reflect a threshold-like pattern, whereby metabolic vulnerability exerts a disproportionate impact once systemic metabolic burden exceeds a higher range, consistent with progressive microvascular injury and limited compensatory capacity (46). In contrast, the absence of a significant overall association with glaucoma may suggest that systemic metabolic vulnerability plays a relatively smaller role than eye-specific factors, such as intraocular pressure, anterior segment anatomy, and optic nerve susceptibility. In addition, any metabolic contribution to glaucoma risk may be heterogeneous across disease subtypes or mediated indirectly through vascular comorbidity rather than acting as a dominant determinant (47). Nevertheless, these proposed pathways remain speculative, and future experimental and mechanistic studies are warranted to clarify causal mechanisms and potential heterogeneity across disease subtypes.

The subgroup findings for DR should be interpreted as exploratory and hypothesis-generating rather than as definitive evidence of effect modification. Within this exploratory framework, the association between higher MVX and incident DR appeared stronger among women, individuals younger than 60 years, and those with obesity. One possible explanation is that sex-related differences in metabolic and inflammatory phenotypes, as well as microvascular susceptibility, may modify how systemic metabolic vulnerability is translated into retinal microvascular injury, potentially contributing to a stronger association in women (48, 49). The age-specific pattern may suggest that elevated MVX in midlife captures earlier or more aggressive metabolic dysregulation that precedes clinically apparent retinopathy, whereas among older adults the relative contribution of MVX may be attenuated by longer disease duration, treatment effects, competing risks, and survival-related selection. Similarly, obesity may amplify this association, as excess adiposity is closely linked to insulin resistance, chronic low-grade inflammation, and endothelial dysfunction, all of which may contribute to retinal microvascular damage (50, 51). However, these interpretations remain tentative and require confirmation in future studies.

Genetic susceptibility represents an important and relatively stable component of risk for age-related eye diseases, and the PRS-based analyses in the present study were intended as secondary exploratory extensions of the main MVX question rather than as the primary focus of our study. In this context, the joint analyses suggest that metabolic vulnerability and genetic susceptibility may provide complementary information at the population level, with individuals exhibiting both high MVX and high genetic risk generally showing the highest relative risks for AMD, cataract, and DR (30, 52). The observed additive interaction for AMD further raises the possibility that systemic metabolic vulnerability and inherited susceptibility may act jointly in shaping disease risk on an absolute scale. However, these findings should be interpreted cautiously. The present study was not designed as a formal prediction study, and we did not evaluate whether adding MVX, PRS, or their combination materially improves discrimination, calibration, reclassification, or clinical decision-making beyond conventional risk factors. Accordingly, these PRS-based and joint analyses should be viewed as exploratory and hypothesis-generating, rather than as evidence of immediate clinical utility for screening or prevention. Additive interaction was evaluated using RERI and AP, which are informative for public health interpretation because they assess whether the excess risk associated with combined exposures exceeds the sum of the excess risks associated with each exposure alone (53).

This study has several strengths. First, analyses were conducted in a large, well-characterized prospective cohort with long-term follow-up, enabling robust estimation of incident risks across multiple age-related eye disease endpoints. Second, MVX was derived from an integrated panel of circulating biomarkers and evaluated using complementary exposure parameterizations, including continuous measures, median-based categories, quartiles, and restricted cubic splines. This approach enhances interpretability and allows assessment of potential non-linearity in associations. Third, genetic susceptibility was assessed using two PRS construction strategies, which allowed a more structured exploratory evaluation of whether metabolic vulnerability and inherited susceptibility may jointly characterize relative risk patterns. Fourth, comprehensive covariate adjustment and exploratory subgroup analyses were applied, and additive interaction was quantified using RERI and AP with bootstrap-derived CIs. Together, these approaches provide an interpretable evaluation of association patterns and joint risk relationships.

Several limitations should be acknowledged. First, selection bias should be considered. Of the 501,939 participants in the full UK Biobank cohort, only 206,311 were included in the present analyses after exclusions, primarily because of missing biomarker data required for MVX calculation and missing covariate information. This substantial restriction may have resulted in an analytic sample that differed systematically from the overall UK Biobank population, thereby limiting generalizability and potentially introducing selection bias. In addition, UK Biobank participants are not fully representative of the general population. Therefore, the findings should be interpreted with caution and validated in other cohorts. Second, outcome ascertainment relied on linked hospital inpatient records from the UK Biobank Hospital Episode Statistics database. As a result, milder or earlier-stage cases managed in outpatient or community settings may not have been captured, which is particularly relevant for conditions such as cataract and early AMD. This may have led to underascertainment of less severe disease, introduced outcome misclassification, and potentially attenuated the observed associations. Third, MVX was assessed at baseline and does not account for longitudinal changes in metabolic vulnerability or the influence of subsequent treatment, which could result in exposure misclassification during follow-up. Fourth, although extensive covariates were included, residual confounding cannot be entirely excluded, and the subgroup and interaction analyses were exploratory and involved multiple comparisons; these findings should therefore be interpreted cautiously. Fifth, the present study was not designed as a formal prediction study. We did not evaluate whether MVX, alone or in combination with PRS, materially improves discrimination, calibration, reclassification, or clinical decision-making beyond conventional risk factors. Therefore, the findings should not be interpreted as establishing immediate clinical utility for diagnosis, screening, or prevention. Sixth, the performance and transferability of PRS may be reduced in populations outside predominantly European ancestry, underscoring the need for external validation in more diverse cohorts. Future studies should seek to replicate these findings in independent populations, evaluate time-varying or repeated measures of metabolic vulnerability, incorporate more detailed ocular phenotypes and clinical measurements, and determine whether MVX, alone or in combination with genetic susceptibility, provides meaningful predictive value beyond conventional risk factors.

## Conclusions

In this prospective UK Biobank cohort, higher metabolic vulnerability, as assessed by MVX, was associated with increased risks of incident AMD, cataract, and DR, but not glaucoma. Secondary exploratory analyses further suggested that metabolic vulnerability and genetic susceptibility may jointly characterize relative risk patterns for some age-related eye diseases, particularly AMD. Together, these findings support the relevance of MVX as a biomarker of metabolic vulnerability associated with several age-related eye diseases at the population level. However, further studies are required to determine whether MVX, alone or in combination with genetic susceptibility, provides meaningful predictive value or clinical utility beyond conventional risk factors.

## Data Availability

The original contributions presented in the study are included in the article/supplementary material, further inquiries can be directed to the corresponding author.
