# Supplementary material for: Metabolic vulnerability, genetic susceptibility, and incident age-related eye diseases: a prospective cohort study
Source: Front Nutr. 2026 Jun 29;13:1840459. doi: 10.3389/fnut.2026.1840459 (PMC13357405; doi:10.3389/fnut.2026.1840459)
Supplement: Supplementary file 1 [file Supplementary_File_1.docx]

**Supplementary file**

Supplementary Methods

Figure S1. Distributions of the IVX across age-related eye diseases.

Figure S2. Distributions of the MMX across age-related eye diseases.

Figure S3. Distributions of the MVX across age-related eye diseases.

Figure S4. PRS-CS and incident age-related eye diseases risk.

Figure S5. Weighted PRS and incident age-related eye diseases risk.

Table S1. Summary of reported susceptibility loci used for the construction of the weighted PRS for AMD.

Table S2. Summary of reported susceptibility loci used for the construction of the weighted PRS for cataract.

Table S3. Summary of reported susceptibility loci used for the construction of the weighted PRS for DR.

Table S4. Summary of reported susceptibility loci used for the construction of the weighted PRS for glaucoma.

Table S5. Coding and definition information of variable in the UK Biobank.

Table S6. Association between PRS and AMD risk.

Table S7. Association between PRS and cataract risk.

Table S8. Association between PRS and DR risk.

Table S9. Association between PRS and glaucoma risk.

Table S10. Additive interaction between MVX and genetic risk in incident age-related eye diseases.

**Supplementary methods**

**Calculation of IVX, MMX, and MVX**

The metabolic vulnerability index (MVX), together with its two component indices, the inflammation vulnerability index (IVX) and the metabolic malnutrition index (MMX), was calculated according to the previously published methodology developed by Otvos et al. in the CATHGEN cohort. MVX is a sex-specific multimarker score derived from six circulating biomarkers quantified by nuclear magnetic resonance (NMR) spectroscopy, including glycoprotein acetyls (GlycA), small high-density lipoprotein particle (sHDL), valine, leucine, isoleucine, and citrate. These biomarkers were identified in the original study as making significant and independent contributions to all-cause mortality prediction in multivariable models. Prior to score construction, each biomarker was winsorized at the 1st and 99th percentiles to reduce the influence of extreme values. Biomarker concentrations were then combined into composite indices using sex-specific regression coefficients derived from mortality prediction models in the CATHGEN cohort. To facilitate comparability, the resulting IVX, MMX, and MVX scores were normalized to a range of 1 to 100. Higher values of IVX, MMX, and MVX indicate greater metabolic vulnerability. The sex-specific equations were defined as follows:

For females:
IVX = 9 − 0.000187 × GlycA − 0.3585 × sHDL + 0.000348 × (GlycA × sHDL)

For males:
IVX = 9 − 0.00437 × GlycA − 0.52307 × sHDL + 0.000817 × (GlycA × sHDL)

MMX reflects metabolic malnutrition and amino acid dysregulation and was derived from valine, leucine, isoleucine, and citrate, including both linear and quadratic terms where applicable. The sex-specific formulas were as follows:

For females:
MMX = [0.353 × (4 − 0.03142 × Leucine + 0.0000893 × Leucine²)]
   + [0.684 × (7 − 0.03362 × Valine + 0.0000689 × Valine²)]
   + 0.00332 × Isoleucine
   + [0.7135 × (1 − 0.0072 × Citrate + 0.0000573 × Citrate²)]

For males:
MMX = [1.076 × (4 − 0.01594 × Leucine + 0.0000291 × Leucine²)]
   + [0.414 × (7 − 0.0239 × Valine + 0.00005 × Valine²)]
   + 0.01265 × Isoleucine
   + [0.5881 × (1 + 0.00906 × Citrate − 0.0000126 × Citrate²)]

MVX was calculated by combining IVX and MMX, including an interaction term to account for the observed synergistic effect between inflammatory and metabolic malnutrition components. The final sex-specific MVX equations were:

For females:
MVX = 2.27278 × IVX + 12.13511 × ln(MMX) − 1.09312 × [IVX × ln(MMX)]

For males:
MVX = 3.54601 × IVX + 14.41428 × ln(MMX) − 1.43438 × [IVX × ln(MMX)]

Higher values of IVX, MMX, and MVX indicate greater metabolic vulnerability. All indices were analyzed as continuous variables and, where appropriate, categorized into quantiles for descriptive and regression analyses.

**Healthy diet**

**“**Healthy diet” was defined using a 10-component food-group checklist. Participants were classified as having a healthy diet if ≥5 of the following 10 criteria were met:

1. Fruit (fresh or dried) ≥ 3 servings/day;
2. Vegetables (cooked, salad, or raw) ≥ 3 servings/day;
3. Whole grains (e.g., whole-meal/whole-grain bread, bran, oat, muesli) ≥ 3 servings/day;
4. Fish (oily or non-oily) ≥ 2 servings/day;
5. Dairy (e.g., milk, cheese) ≥ 2 servings/day;
6. Vegetable oils (e.g., olive-oil–based, sunflower/polyunsaturated oil–based, soft margarine, low/reduced-fat spread) ≥ 2 servings/day;
7. Processed meats ≤ 1 serving/day;
8. Unprocessed red meats (poultry, beef, lamb/mutton, pork) ≤ 2 servings/day;
9. Refined grains (white/brown bread, other breads, biscuits, other cereals) ≤ 2 servings/day;
10. No consumption of sugar-sweetened beverages.

**
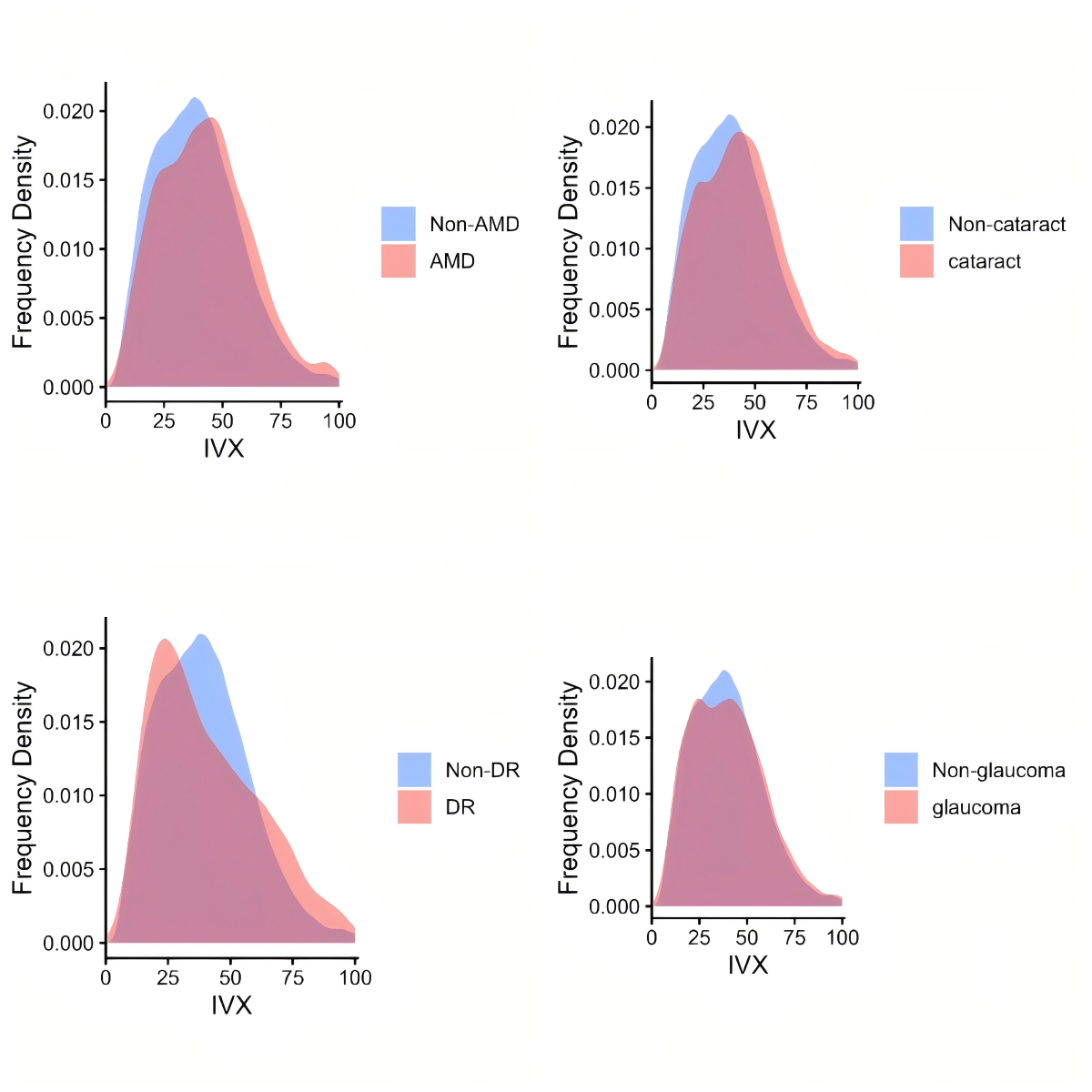
**

Figure S1. Distributions of the inflammation vulnerability index (IVX) across age-related eye diseases. Kernel density plots compare IVX distributions between participants with and without age-related macular degeneration (AMD), cataract, diabetic retinopathy (DR), and glaucoma.

**
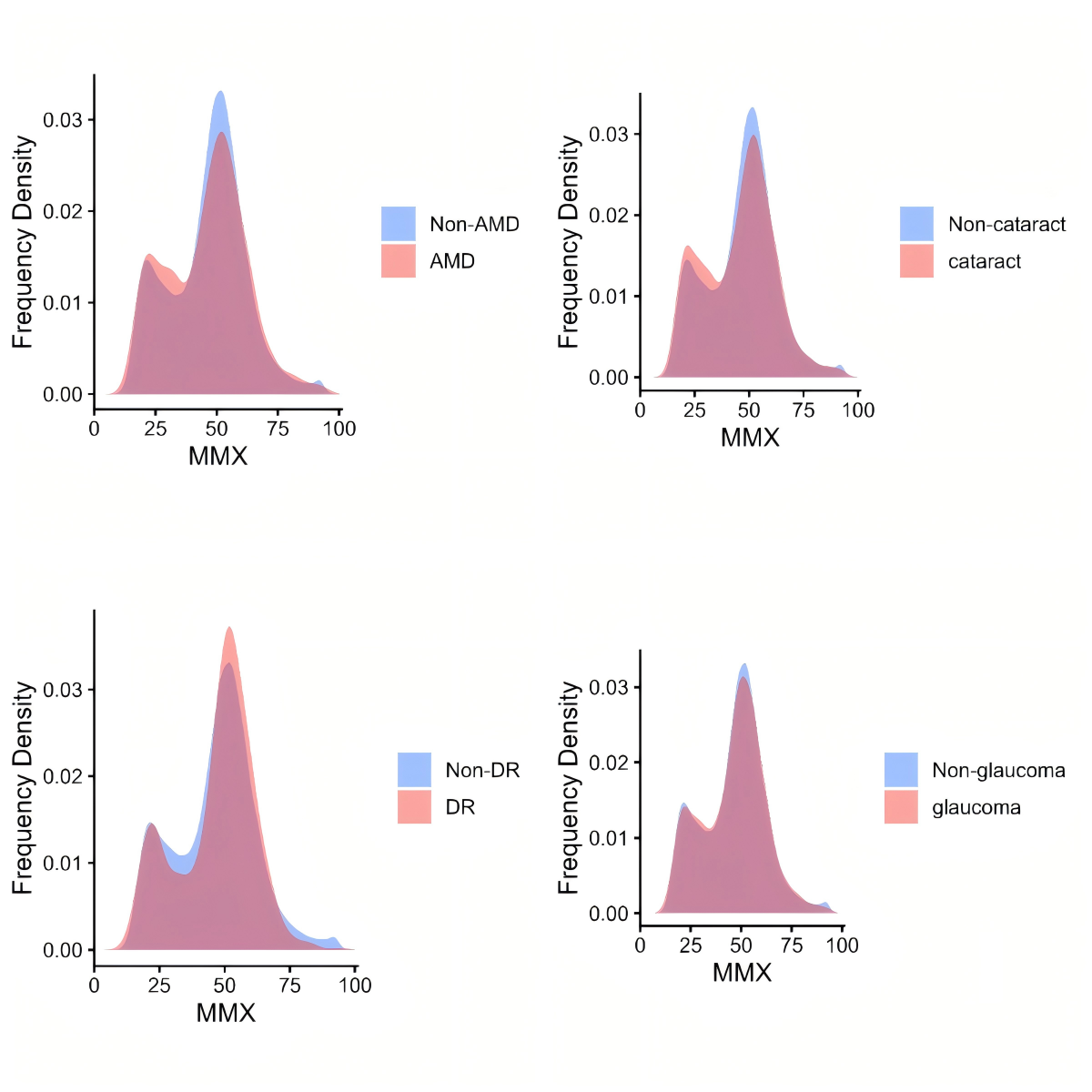
**

Figure S2. Distributions of the metabolic malnutrition index (MMX) across age-related eye diseases. Kernel density plots compare MMX distributions between participants with and without age-related macular degeneration (AMD), cataract, diabetic retinopathy (DR), and glaucoma.

**
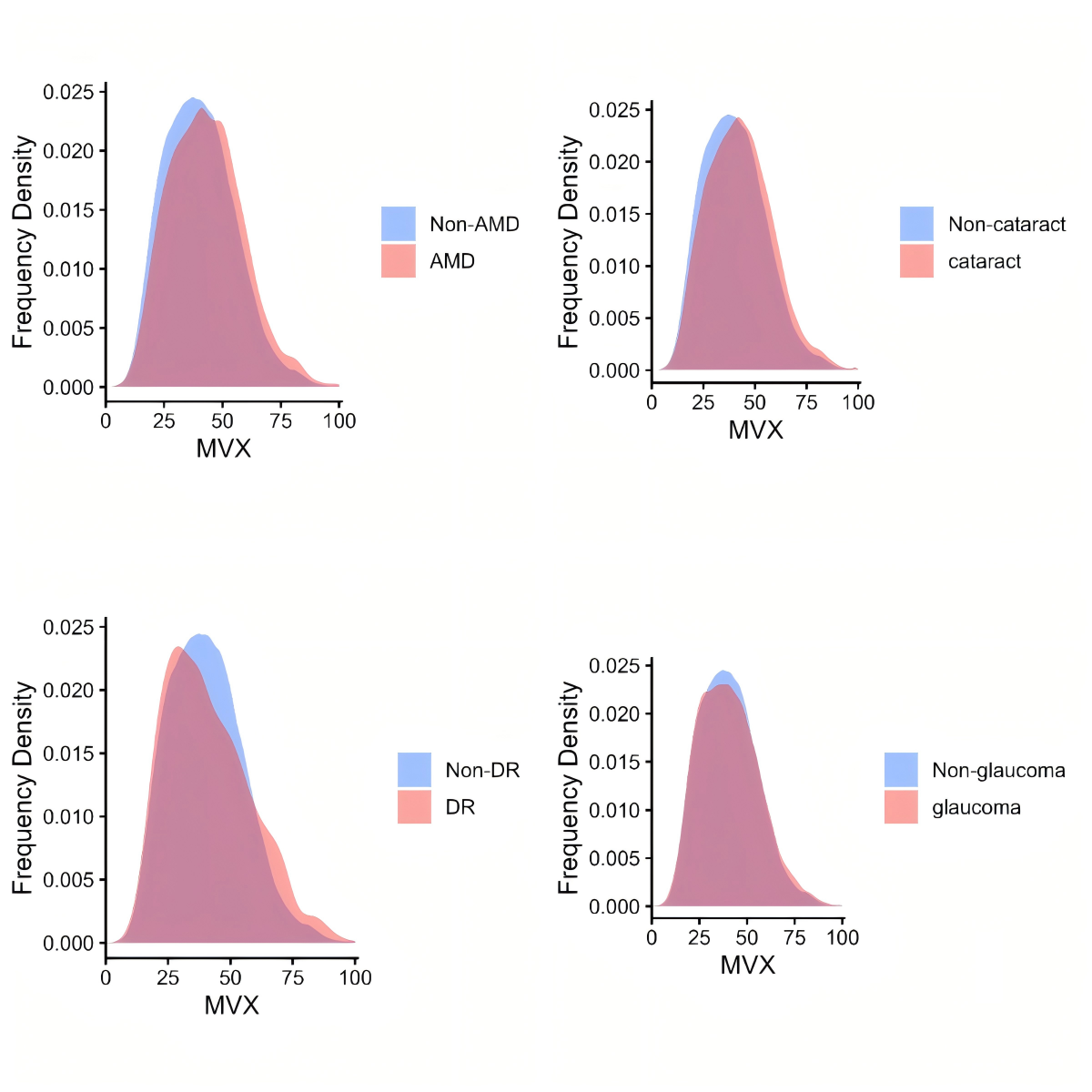
**

Figure S3. Distributions of the metabolic vulnerability index (MVX) across age-related eye diseases. Kernel density plots compare MVX distributions between participants with and without age-related macular degeneration (AMD), cataract, diabetic retinopathy (DR), and glaucoma.


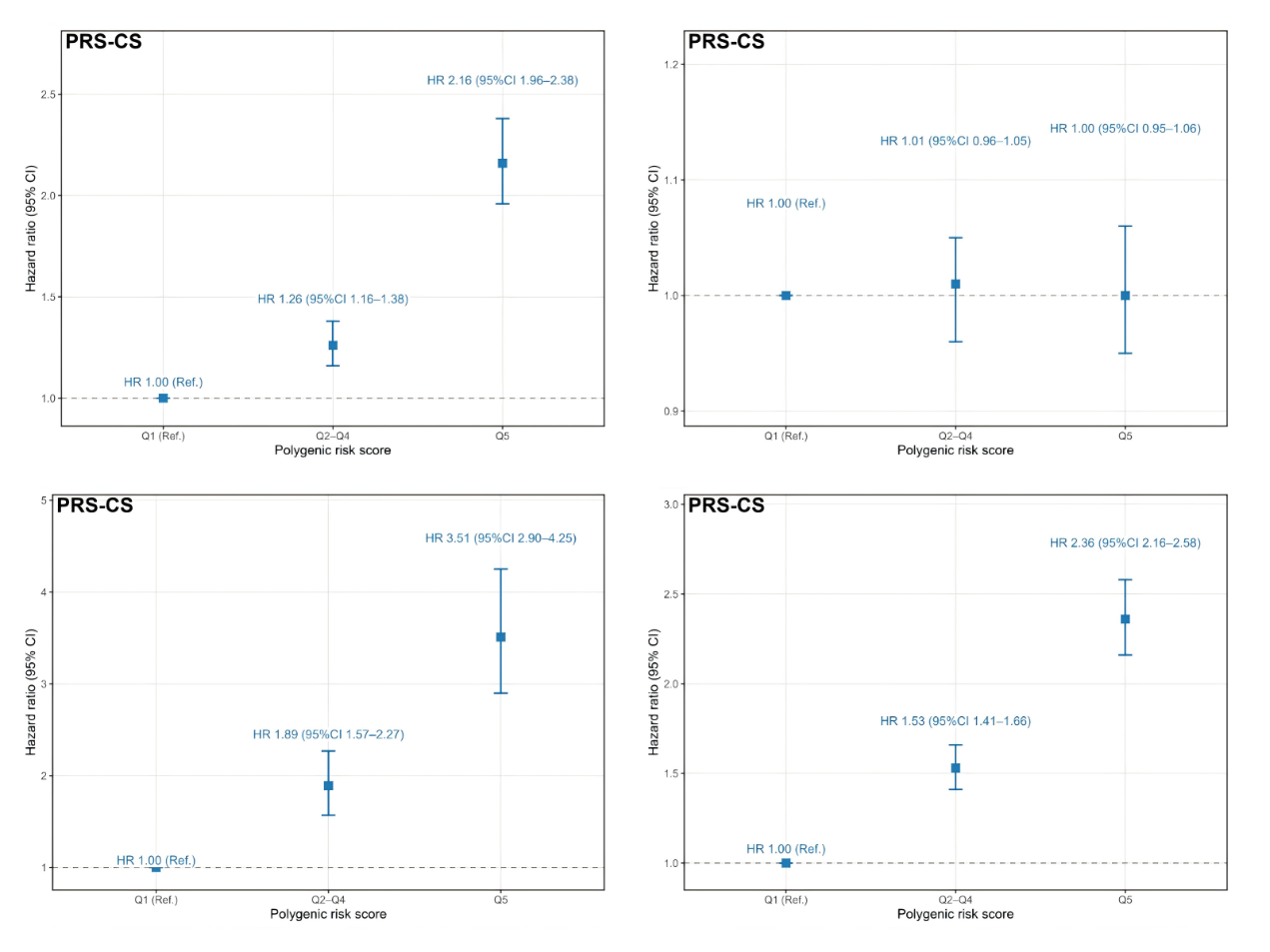


Figure S4. PRS-CS and incident age-related eye diseases risk. (Q1 as reference) Chronological age, sex, ethnicity, education, BMI, and Townsend deprivation index, physical activity, sleep duration, smoking, alcohol consumption, and healthy diet were adjusted in the analyses. PRS: polygenic risk score; CS: continuous shrinkage; AMD: age-related macular degeneration; DR: diabetic retinopathy.


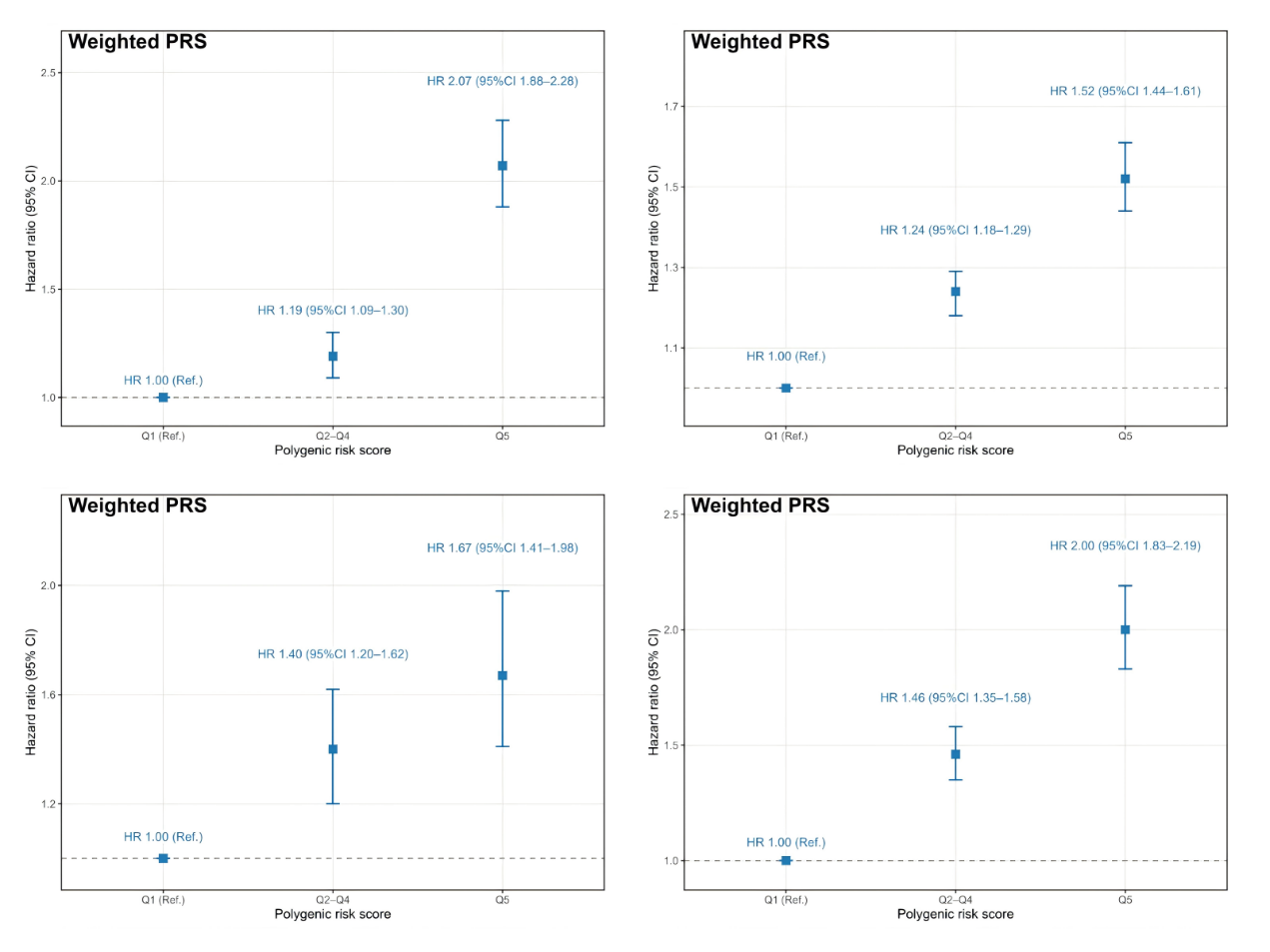


Figure S5. Weighted PRS and incident age-related eye diseases risk. (Q1 as reference) Chronological age, sex, ethnicity, education, BMI, and Townsend deprivation index, physical activity, sleep duration, smoking, alcohol consumption, and healthy diet were adjusted in the analyses. PRS: polygenic risk score; AMD: age-related macular degeneration; DR: diabetic retinopathy.

Table S1. Summary of reported susceptibility loci used for the construction of the weighted PRS for AMD.

| RSID | CHR | BP | Effect | Weight |
| --- | --- | --- | --- | --- |
| rs10458561 | 1 | 70921173 | A | 1.130032E-04 |
| rs10801555 | 1 | 196660261 | G | -1.011573E-01 |
| rs10737680 | 1 | 196679455 | C | -2.847935E-03 |
| rs1329428 | 1 | 196702810 | T | -4.201819E-02 |
| rs7603418 | 2 | 57723162 | G | 1.093565E-03 |
| rs17585147 | 2 | 59686346 | G | 5.897250E-04 |
| rs2729287 | 3 | 2137538 | G | -7.512161E-04 |
| rs17736733 | 5 | 169107017 | T | -5.509217E-03 |
| rs1042663 | 6 | 31905130 | A | -9.084440E-03 |
| rs429608 | 6 | 31930462 | A | -2.844124E-02 |
| rs1677499 | 7 | 137584781 | G | -1.503716E-04 |
| rs243538 | 7 | 148404379 | A | 3.834191E-04 |
| rs10490924 | 10 | 124214448 | T | 2.095018E-01 |
| rs2014307 | 10 | 124217632 | G | 7.858621E-04 |
| rs4760980 | 12 | 128062971 | C | -1.902392E-03 |
| rs10048146 | 16 | 86710660 | G | 2.873923E-02 |
| rs4790589 | 17 | 1447756 | T | 1.616816E-03 |
| rs10502721 | 18 | 36860656 | A | 4.474555E-04 |
| rs2181874 | 20 | 52784478 | A | 3.799269E-03 |

RSID: variant identifier; CHR: chromosome; BP: base-pair position; Effect: effect allele used for scoring; Weight: per-SNP weight in the PRS; PRS: polygenic risk score; AMD: age-related macular degeneration.

Table S2. Summary of reported susceptibility loci used for the construction of the weighted PRS for cataract.

| RSID | CHR | BP | Effect | Weight |
| --- | --- | --- | --- | --- |
| rs10910013 | 1 | 3613770 | T | 9.992801E-05 |
| rs7552012 | 1 | 8393145 | T | 2.738899E-03 |
| rs2999867 | 1 | 13890695 | C | -1.600877E-03 |
| rs10737914 | 1 | 14210730 | T | 4.673536E-03 |
| rs2863458 | 1 | 16389026 | C | 3.810912E-03 |
| rs10799824 | 1 | 19841174 | A | 2.833825E-03 |
| rs6658127 | 1 | 21875390 | A | 4.459853E-03 |
| rs2275363 | 1 | 21940555 | T | -4.935543E-04 |
| rs6678992 | 1 | 22453842 | C | 2.441488E-03 |
| rs2072747 | 1 | 26098107 | A | 5.591359E-03 |
| rs4653301 | 1 | 38028374 | T | 1.201208E-03 |
| rs4233215 | 1 | 38098036 | A | 1.420567E-03 |
| rs2455637 | 1 | 50843776 | C | 3.866121E-03 |
| rs2500452 | 1 | 51458372 | G | -2.154899E-02 |
| rs17398598 | 1 | 52024908 | C | -4.340161E-03 |
| rs1655519 | 1 | 55119515 | G | 2.024887E-03 |
| rs17399810 | 1 | 55119534 | T | -1.755732E-02 |
| rs2764689 | 1 | 58482203 | T | 1.752782E-03 |
| rs2989476 | 1 | 61059259 | C | 5.782523E-03 |
| rs1473753 | 1 | 62849627 | C | 6.529924E-03 |
| rs1184626 | 1 | 77093180 | A | -2.630267E-03 |
| rs9324189 | 1 | 82239042 | G | -3.855590E-03 |
| rs7521759 | 1 | 97099941 | C | 3.970297E-03 |
| rs4950124 | 1 | 98875469 | T | -1.623011E-03 |
| rs11588761 | 1 | 106870651 | C | 2.245071E-04 |
| rs12411132 | 1 | 112477712 | A | -2.034734E-03 |
| rs10923551 | 1 | 118886721 | C | 2.552347E-03 |
| rs4970851 | 1 | 145535611 | G | -1.980746E-03 |
| rs7536659 | 1 | 151816718 | C | -4.521961E-03 |
| rs843971 | 1 | 153277423 | T | 4.324338E-03 |
| rs9426902 | 1 | 153718746 | A | 1.823641E-04 |
| rs12044132 | 1 | 154462360 | T | 1.883119E-02 |
| rs1934082 | 1 | 159991622 | G | -1.449971E-03 |
| rs7527826 | 1 | 161969322 | A | 3.243957E-03 |
| rs6674132 | 1 | 162611515 | A | -1.907903E-03 |
| rs12141039 | 1 | 177081676 | C | 5.861064E-04 |
| rs3131332 | 1 | 177740608 | T | -8.701519E-03 |
| rs3814341 | 1 | 184596806 | G | 6.268609E-03 |
| rs34430818 | 1 | 187016166 | C | -5.592969E-03 |
| rs12025826 | 1 | 192940642 | A | -9.790128E-03 |
| rs2990510 | 1 | 197020658 | G | 3.851373E-03 |
| rs10900520 | 1 | 203780591 | G | -6.213255E-03 |
| rs7550796 | 1 | 204054323 | A | 1.718857E-04 |
| rs867408 | 1 | 217158960 | G | -3.187825E-03 |
| rs17048367 | 1 | 218833890 | G | -4.852173E-03 |
| rs6667260 | 1 | 226923938 | C | 6.795402E-03 |
| rs12734075 | 1 | 229693132 | C | 9.824224E-04 |
| rs11122548 | 1 | 230725969 | A | -1.498846E-03 |
| rs4659682 | 1 | 236682248 | G | -1.094632E-03 |
| rs578476 | 1 | 241178009 | T | -3.022671E-03 |
| rs1635517 | 1 | 242012033 | A | -1.377055E-02 |
| rs10926992 | 1 | 243494739 | G | 2.017132E-03 |
| rs12075565 | 1 | 247614194 | G | -7.752494E-04 |
| rs869111 | 1 | 247752367 | G | -1.984509E-03 |
| rs12240276 | 10 | 4889403 | T | -2.024101E-02 |
| rs11253156 | 10 | 5437365 | G | -3.366536E-03 |
| rs12765374 | 10 | 5644621 | A | -2.470973E-03 |
| rs7909299 | 10 | 5685301 | T | -1.717410E-03 |
| rs45575338 | 10 | 5784151 | G | -3.257238E-03 |
| rs2380205 | 10 | 5886734 | T | -2.761180E-03 |
| rs35724595 | 10 | 6767306 | A | 4.785001E-03 |
| rs11255148 | 10 | 7511562 | G | 1.132420E-02 |
| rs41298373 | 10 | 7622009 | A | 8.034863E-03 |
| rs17148786 | 10 | 10789461 | T | 7.198227E-04 |
| rs34445355 | 10 | 13776656 | A | 3.003780E-03 |
| rs780825 | 10 | 16912134 | A | 1.759171E-03 |
| rs2230469 | 10 | 22839628 | C | -7.246353E-03 |
| rs12250715 | 10 | 28685674 | T | -1.283308E-04 |
| rs11815517 | 10 | 28699067 | G | -1.359717E-03 |
| rs1413792 | 10 | 29016275 | C | -9.365665E-04 |
| rs1775715 | 10 | 32309005 | G | -1.193533E-02 |
| rs1325488 | 10 | 44448756 | T | -2.585809E-04 |
| rs2590381 | 10 | 49578487 | C | -9.547103E-03 |
| rs7912792 | 10 | 60335179 | T | -1.075382E-02 |
| rs10509543 | 10 | 90072352 | C | -2.415947E-03 |
| rs1935961 | 10 | 95783242 | A | 4.236374E-03 |
| rs2274224 | 10 | 96039597 | C | 2.386271E-02 |
| rs549062 | 10 | 97222023 | A | -5.112329E-03 |
| rs7905661 | 10 | 99570779 | G | -9.460829E-04 |
| rs1885301 | 10 | 101541053 | G | -1.159352E-03 |
| rs11190305 | 10 | 101639877 | C | 9.183633E-03 |
| rs12785223 | 10 | 104844011 | G | -2.305426E-03 |
| rs4506565 | 10 | 114756041 | T | 7.188637E-03 |
| rs10749138 | 10 | 115370274 | C | 1.406070E-03 |
| rs2227310 | 10 | 115489152 | G | 1.186337E-02 |
| rs4752681 | 10 | 124066189 | A | 7.036071E-03 |
| rs10490924 | 10 | 124214448 | T | 6.062507E-03 |
| rs1891110 | 10 | 124610027 | A | -4.187203E-03 |
| rs3781412 | 10 | 126715154 | G | 5.494545E-03 |
| rs4962311 | 10 | 127244526 | C | 3.882508E-03 |
| rs10764993 | 10 | 132659862 | C | -2.434309E-03 |
| rs2818387 | 10 | 133954011 | T | 1.263209E-02 |
| rs11605246 | 11 | 198062 | G | -4.564148E-03 |
| rs7116130 | 11 | 244129 | T | 1.145530E-03 |
| rs3210908 | 11 | 406483 | C | 3.243450E-03 |
| rs12276847 | 11 | 2323022 | T | -2.368131E-03 |
| rs2647543 | 11 | 5496811 | C | 1.807736E-03 |
| rs2055709 | 11 | 7272397 | T | -2.339533E-03 |
| rs4376885 | 11 | 7503395 | C | 5.748915E-03 |
| rs11022079 | 11 | 11913585 | T | -8.983287E-03 |
| rs2925145 | 11 | 18257123 | C | 6.257661E-03 |
| rs74718371 | 11 | 30848413 | T | -1.523628E-02 |
| rs10836202 | 11 | 34402778 | T | 3.922503E-05 |
| rs178501 | 11 | 44081716 | A | -1.590005E-03 |
| rs1459101 | 11 | 55339652 | T | 3.056955E-03 |
| rs72003051 | 11 | 56380546 | C | -1.103598E-03 |
| rs7103033 | 11 | 57983194 | G | -7.179552E-03 |
| rs2469887 | 11 | 60704213 | T | 2.548491E-02 |
| rs10792367 | 11 | 62758799 | G | -4.174882E-03 |
| rs61886888 | 11 | 64124622 | A | -2.933881E-03 |
| rs648336 | 11 | 68616449 | T | 2.659196E-03 |
| rs10896380 | 11 | 68682402 | G | -4.990834E-03 |
| rs622082 | 11 | 68703959 | G | 7.686874E-03 |
| rs12574505 | 11 | 68797752 | G | 3.201945E-03 |
| rs74342245 | 11 | 68938776 | A | 4.477503E-02 |
| rs4044287 | 11 | 74267187 | C | 8.503718E-03 |
| rs610183 | 11 | 94807581 | G | -3.013112E-03 |
| rs640455 | 11 | 94812057 | G | -6.335085E-04 |
| rs1939309 | 11 | 100421331 | T | 7.379928E-04 |
| rs2070661 | 11 | 108043988 | T | -1.437626E-04 |
| rs573455 | 11 | 117267884 | G | -7.139920E-03 |
| rs7947524 | 11 | 117671091 | T | -8.735452E-04 |
| rs2512227 | 11 | 124056732 | G | -2.326891E-03 |
| rs657361 | 11 | 126111091 | A | 1.546998E-03 |
| rs71447498 | 12 | 664494 | A | 1.501125E-02 |
| rs4766080 | 12 | 3368198 | C | 1.269005E-03 |
| rs6489457 | 12 | 3449155 | G | -2.564049E-03 |
| rs7306434 | 12 | 8045597 | A | -2.436488E-03 |
| rs6488767 | 12 | 8057859 | T | -1.382872E-03 |
| rs12821106 | 12 | 13576211 | A | -7.881378E-04 |
| rs11056989 | 12 | 16687223 | T | -9.214023E-03 |
| rs111626763 | 12 | 26834804 | TACTC | 1.904272E-03 |
| rs753013 | 12 | 29052910 | A | 3.567402E-03 |
| rs7309866 | 12 | 29244346 | T | -8.355009E-03 |
| rs2304630 | 12 | 30881809 | C | -8.615816E-03 |
| rs2074533 | 12 | 48134695 | C | -1.271524E-03 |
| rs2658658 | 12 | 52788928 | A | -1.018168E-03 |
| rs58036029 | 12 | 55523586 | A | -1.815508E-03 |
| rs7297618 | 12 | 63142592 | C | -3.310026E-03 |
| rs74552226 | 12 | 91893488 | T | 8.176600E-04 |
| rs1598943 | 12 | 91912198 | T | 3.548216E-03 |
| rs11108101 | 12 | 95941907 | A | -4.795432E-03 |
| rs11111315 | 12 | 102983914 | A | 2.434778E-03 |
| rs7298565 | 12 | 109937534 | A | 7.414679E-03 |
| rs78776661 | 12 | 119983754 | G | -2.164756E-03 |
| rs1384556 | 12 | 125579680 | G | -1.867490E-03 |
| rs7133864 | 12 | 125606180 | T | 1.821493E-03 |
| rs10846838 | 12 | 125652226 | G | 2.562927E-03 |
| rs264505 | 12 | 130163457 | T | 1.429865E-04 |
| rs2104324 | 13 | 20692349 | A | -5.042164E-04 |
| rs1467719 | 13 | 31054166 | G | -1.675231E-03 |
| rs1535681 | 13 | 43329467 | T | -3.047602E-03 |
| rs3742264 | 13 | 46648094 | T | 5.016520E-03 |
| rs9596497 | 13 | 51882372 | A | 3.815242E-03 |
| rs9536430 | 13 | 54060972 | T | -7.079131E-04 |
| rs12875327 | 13 | 75314681 | C | -4.838771E-03 |
| rs9573518 | 13 | 75885355 | C | -3.169902E-03 |
| rs12867658 | 13 | 78006371 | G | -5.158565E-03 |
| rs504122 | 13 | 80911525 | A | -1.404442E-02 |
| rs1334199 | 13 | 89195892 | G | 5.543519E-04 |
| rs9584792 | 13 | 98920453 | C | 2.266413E-03 |
| rs1058083 | 13 | 100038233 | G | 1.984051E-03 |
| rs9585824 | 13 | 102627889 | G | 3.412467E-04 |
| rs1337935 | 13 | 109550246 | G | 9.191193E-03 |
| rs914023 | 13 | 113424393 | G | 1.980274E-03 |
| rs10146120 | 14 | 22114604 | G | -2.142152E-03 |
| rs2178779 | 14 | 22409571 | G | -3.946358E-03 |
| rs1271806 | 14 | 25629226 | A | -4.457950E-03 |
| rs4981820 | 14 | 31531855 | C | 2.960949E-04 |
| rs34644576 | 14 | 32385747 | T | -5.461357E-04 |
| rs1579702 | 14 | 33817553 | G | -2.539266E-03 |
| rs996347 | 14 | 34410892 | C | 8.315745E-03 |
| rs7143346 | 14 | 35700562 | T | 3.223354E-04 |
| rs2355654 | 14 | 50455087 | C | 3.670209E-03 |
| rs762063 | 14 | 53098902 | A | -3.949038E-03 |
| rs927540 | 14 | 58049900 | G | -6.535296E-03 |
| rs111562516 | 14 | 58669659 | A | 3.389917E-03 |
| rs1051858 | 14 | 58831142 | G | -2.231228E-03 |
| rs12586711 | 14 | 60932752 | A | -5.629186E-03 |
| rs4396463 | 14 | 77588882 | C | -2.661298E-03 |
| rs979322 | 14 | 85048815 | C | -2.079158E-03 |
| rs1048755 | 14 | 92548785 | T | -2.234003E-03 |
| rs7150379 | 14 | 92736604 | C | -5.242663E-03 |
| rs7161072 | 14 | 93787360 | T | -1.249704E-03 |
| rs2209806 | 14 | 97641791 | A | 2.576151E-03 |
| rs7157599 | 14 | 100625902 | T | -1.232348E-03 |
| rs12589075 | 14 | 101644828 | G | -6.828157E-03 |
| rs9972231 | 14 | 105615648 | T | 2.863328E-03 |
| rs10152498 | 15 | 24123835 | G | 3.324178E-03 |
| rs12442344 | 15 | 26037922 | T | 1.877335E-03 |
| rs1800407 | 15 | 28230318 | T | 1.372683E-02 |
| rs2676097 | 15 | 33759652 | A | -8.255397E-03 |
| rs2036814 | 15 | 36230452 | G | -1.263718E-03 |
| rs11858113 | 15 | 40914177 | C | 1.753317E-03 |
| rs28434362 | 15 | 41214152 | T | -1.005543E-02 |
| rs11071334 | 15 | 57878811 | C | 7.406670E-03 |
| rs2100431 | 15 | 60770850 | A | 1.736128E-03 |
| rs4461027 | 15 | 74211548 | T | 1.725972E-03 |
| rs893817 | 15 | 74229065 | A | -4.071261E-03 |
| rs2289524 | 15 | 78390414 | C | 4.158708E-03 |
| rs11631733 | 15 | 80271752 | G | 4.392245E-03 |
| rs57144603 | 15 | 80560548 | G | -1.686240E-03 |
| rs11630197 | 15 | 82574879 | C | 3.363853E-03 |
| rs2280465 | 15 | 89417629 | A | 1.000919E-03 |
| rs1457851 | 15 | 95524439 | A | -1.845754E-04 |
| rs12900283 | 15 | 95926961 | T | -4.096963E-03 |
| rs10852058 | 15 | 97511405 | G | -8.412020E-03 |
| rs8017 | 16 | 2821573 | T | 2.205059E-03 |
| rs11864324 | 16 | 4281391 | G | 1.314643E-03 |
| rs11642971 | 16 | 7255857 | G | 7.409263E-03 |
| rs12051285 | 16 | 7460115 | G | -1.718423E-02 |
| rs1070502 | 16 | 10265157 | T | 6.626982E-04 |
| rs11863884 | 16 | 16946710 | T | -1.750019E-03 |
| rs13334665 | 16 | 18125091 | C | -2.759931E-03 |
| rs12444661 | 16 | 19247797 | A | -1.604289E-03 |
| rs35873068 | 16 | 27064137 | T | -1.136786E-03 |
| rs34929254 | 16 | 50455030 | A | -2.135443E-03 |
| rs4783865 | 16 | 55069693 | A | -1.024177E-04 |
| rs12925700 | 16 | 69793463 | A | -1.992283E-02 |
| rs4985446 | 16 | 69880293 | C | 7.514598E-04 |
| rs6499270 | 16 | 69917740 | T | 4.157133E-03 |
| rs8043589 | 16 | 73217601 | T | 4.259708E-03 |
| rs10514484 | 16 | 80162126 | G | -3.097379E-03 |
| rs7189799 | 16 | 80337163 | C | -1.707858E-03 |
| rs3866569 | 16 | 85887693 | T | 2.260878E-03 |
| rs2696860 | 16 | 86327721 | G | 1.632348E-03 |
| rs8050937 | 16 | 86389680 | A | 2.487426E-03 |
| rs10048146 | 16 | 86710660 | G | 1.488692E-02 |
| rs4968145 | 17 | 455814 | A | 4.257250E-03 |
| rs7224258 | 17 | 1255502 | C | 3.496553E-03 |
| rs12103631 | 17 | 1886817 | A | 5.150881E-03 |
| rs12938775 | 17 | 2574821 | A | 1.614606E-03 |
| rs2028000 | 17 | 2706593 | C | -9.919903E-03 |
| rs887389 | 17 | 3793303 | G | -3.119480E-03 |
| rs7215121 | 17 | 4461748 | A | 5.879566E-04 |
| rs2289642 | 17 | 6515387 | G | 9.340662E-04 |
| rs9892297 | 17 | 7446027 | G | -4.848684E-03 |
| rs4968210 | 17 | 7457352 | A | 6.572791E-04 |
| rs727428 | 17 | 7537792 | C | 3.884980E-03 |
| rs3027300 | 17 | 7988993 | C | 9.050727E-04 |
| rs2030793 | 17 | 8990901 | G | 8.909036E-03 |
| rs17499261 | 17 | 10774278 | A | -8.998849E-03 |
| rs34521870 | 17 | 10787384 | T | 2.488427E-03 |
| rs2969195 | 17 | 11429715 | A | 4.071763E-03 |
| rs2072279 | 17 | 13980350 | A | -8.054724E-04 |
| rs712270 | 17 | 18057167 | T | -2.462835E-03 |
| rs12939740 | 17 | 27535054 | C | -9.975424E-03 |
| rs2041156 | 17 | 27568780 | C | -2.332120E-03 |
| rs2289629 | 17 | 27959903 | A | 9.156759E-04 |
| rs8079471 | 17 | 30218317 | T | 2.533444E-03 |
| rs10852948 | 17 | 36648503 | G | 8.017113E-03 |
| rs9908084 | 17 | 42987523 | A | -5.600865E-04 |
| rs9916491 | 17 | 42987524 | C | -5.081979E-03 |
| rs242557 | 17 | 44019712 | A | -2.035031E-03 |
| rs4968318 | 17 | 45451894 | A | 2.092610E-03 |
| rs61051048 | 17 | 47211515 | G | -9.230068E-03 |
| rs2693521 | 17 | 49834814 | C | -4.762502E-03 |
| rs35199094 | 17 | 50341661 | T | 1.642763E-03 |
| rs6504907 | 17 | 52482930 | A | -4.277962E-03 |
| rs3744108 | 17 | 56585872 | C | -1.774576E-04 |
| rs1292053 | 17 | 57963537 | G | 2.668444E-04 |
| rs2676289 | 17 | 62705738 | A | -2.880332E-04 |
| rs887797 | 17 | 64579445 | A | -6.776605E-05 |
| rs1558876 | 17 | 66364691 | G | 1.175510E-02 |
| rs180160 | 17 | 68026021 | T | -7.180732E-04 |
| rs219937 | 17 | 68318758 | T | -3.760919E-03 |
| rs2430514 | 17 | 69676169 | C | -8.543112E-04 |
| rs9913611 | 17 | 69995886 | T | -3.934594E-03 |
| rs1878061 | 17 | 72610089 | T | -1.137130E-03 |
| rs11871056 | 17 | 75164886 | G | 9.956385E-03 |
| rs94598 | 17 | 75385269 | T | 4.200370E-03 |
| rs7223467 | 17 | 81051887 | G | 1.919065E-02 |
| rs563155 | 18 | 166819 | C | 1.833765E-03 |
| rs2856966 | 18 | 907710 | G | 1.174713E-02 |
| rs9646483 | 18 | 1741935 | G | -4.378004E-03 |
| rs11876399 | 18 | 3615943 | T | -1.146974E-03 |
| rs10401115 | 18 | 4107401 | A | 5.696026E-03 |
| rs56104518 | 18 | 6441958 | T | 3.388631E-03 |
| rs2110540 | 18 | 10151888 | T | -1.549293E-03 |
| rs12605289 | 18 | 10181500 | C | 4.670299E-03 |
| rs10468691 | 18 | 13548548 | T | 6.149758E-04 |
| rs1560851 | 18 | 20798766 | T | 1.465454E-03 |
| rs6508344 | 18 | 22646735 | A | -4.445733E-04 |
| rs230655 | 18 | 24485533 | T | 4.911207E-03 |
| rs949216 | 18 | 32422326 | C | -4.246483E-03 |
| rs2625065 | 18 | 33913498 | A | -1.694011E-03 |
| rs563563 | 18 | 34272445 | G | 4.512100E-05 |
| rs9948382 | 18 | 45509964 | G | 6.842874E-03 |
| rs12709758 | 18 | 49479761 | G | -3.649948E-03 |
| rs1788234 | 18 | 67566587 | T | -2.990554E-03 |
| rs6566546 | 18 | 69297624 | A | 8.757404E-03 |
| rs1942370 | 18 | 69308056 | A | 1.639111E-03 |
| rs350156 | 19 | 912852 | C | 8.497224E-03 |
| rs4807072 | 19 | 1320149 | A | 1.487184E-03 |
| rs17685098 | 19 | 2396611 | T | 8.382902E-03 |
| rs3764585 | 19 | 2520791 | A | 1.678663E-03 |
| rs4806907 | 19 | 3093163 | C | 6.873150E-04 |
| rs461645 | 19 | 6919753 | G | 1.664968E-05 |
| rs612862 | 19 | 7593589 | T | 1.822877E-03 |
| rs577029 | 19 | 7624335 | T | 2.619781E-03 |
| rs1644757 | 19 | 7663768 | G | -4.017737E-03 |
| rs2303164 | 19 | 8122737 | A | 3.504967E-03 |
| rs2288904 | 19 | 10742170 | G | 1.044656E-02 |
| rs1054486 | 19 | 12774208 | C | 1.363620E-02 |
| rs6512265 | 19 | 18502835 | A | 1.613031E-03 |
| rs3177137 | 19 | 19023853 | C | -2.533969E-03 |
| rs7252798 | 19 | 21908053 | T | -2.309437E-03 |
| rs62104111 | 19 | 30639831 | T | -2.588706E-03 |
| rs10405693 | 19 | 45326664 | T | -1.748231E-04 |
| rs3178166 | 19 | 45594170 | G | -8.160882E-04 |
| rs11672271 | 19 | 45619946 | G | -1.825592E-03 |
| rs2286756 | 19 | 46145025 | T | 1.180150E-02 |
| rs311370 | 19 | 47446691 | G | 1.316860E-03 |
| rs3745762 | 19 | 48184474 | T | 4.872985E-03 |
| rs4802867 | 19 | 52299885 | G | 1.820203E-03 |
| rs13382164 | 19 | 53304563 | C | -5.107104E-05 |
| rs2074858 | 19 | 57723007 | C | 9.335740E-03 |
| rs201340833 | 19 | 58017752 | G | 7.758375E-03 |
| rs1448574 | 2 | 6438598 | G | 1.975701E-04 |
| rs11883561 | 2 | 16059499 | C | -1.552275E-03 |
| rs1365971 | 2 | 16199568 | G | 5.530962E-03 |
| rs4567937 | 2 | 18676265 | A | 4.011317E-03 |
| rs17442753 | 2 | 19646090 | A | -1.109342E-03 |
| rs10186023 | 2 | 28849435 | C | 5.735457E-05 |
| rs7586405 | 2 | 28906644 | G | 1.230798E-02 |
| rs7600979 | 2 | 36854548 | T | 1.400795E-02 |
| rs4670768 | 2 | 37946055 | G | -5.215285E-03 |
| rs1158411 | 2 | 43793725 | A | 4.351566E-03 |
| rs884399 | 2 | 45982925 | T | 4.356825E-03 |
| rs4953420 | 2 | 47012594 | A | -6.527740E-03 |
| rs4324359 | 2 | 48253268 | G | 4.782828E-03 |
| rs2355803 | 2 | 52335664 | G | -7.370471E-03 |
| rs76285729 | 2 | 56403955 | G | -8.404630E-03 |
| rs34078384 | 2 | 57262343 | T | 2.019401E-03 |
| rs6723710 | 2 | 58824738 | A | -1.496574E-03 |
| rs847803 | 2 | 64982415 | G | 1.643814E-02 |
| rs2861111 | 2 | 66819030 | G | -1.058659E-04 |
| rs11675077 | 2 | 66902745 | A | 3.632381E-03 |
| rs2272051 | 2 | 74007136 | C | 7.365259E-05 |
| rs4247303 | 2 | 85554080 | A | 2.386190E-03 |
| rs66468243 | 2 | 99226172 | A | 4.071476E-03 |
| rs13411997 | 2 | 161672615 | C | 3.096409E-03 |
| rs2298771 | 2 | 166892788 | T | -1.175830E-02 |
| rs3769769 | 2 | 170675765 | T | 2.066988E-05 |
| rs4850350 | 2 | 193443247 | T | -6.459794E-04 |
| rs1864352 | 2 | 209510934 | A | 6.024783E-03 |
| rs804340 | 2 | 217256027 | C | -1.720775E-03 |
| rs10490765 | 2 | 218356528 | C | 1.836204E-02 |
| rs34681466 | 2 | 218416322 | C | 3.691667E-03 |
| rs1550094 | 2 | 233385396 | A | -6.382311E-03 |
| rs1881492 | 2 | 233406998 | G | -9.906239E-03 |
| rs2361503 | 2 | 234704318 | G | -5.121865E-03 |
| rs2219062 | 2 | 237149375 | T | 1.913231E-03 |
| rs13384601 | 2 | 238522813 | A | 7.694078E-03 |
| rs28533806 | 2 | 239832682 | C | -3.560367E-03 |
| rs6749416 | 2 | 240686946 | G | 2.785079E-04 |
| rs6437353 | 2 | 241558397 | A | -4.868404E-03 |
| rs13006939 | 2 | 242255830 | T | 8.379109E-03 |
| rs4247179 | 2 | 242585616 | A | -1.771779E-03 |
| rs480789 | 20 | 932618 | C | -7.091795E-03 |
| rs2853224 | 20 | 3523756 | C | -9.948405E-05 |
| rs1715373 | 20 | 4867140 | G | -1.680751E-03 |
| rs6038044 | 20 | 5024338 | A | 5.620684E-03 |
| rs6053384 | 20 | 5406093 | G | -4.024593E-03 |
| rs2073290 | 20 | 13830137 | C | 1.618573E-02 |
| rs6044267 | 20 | 16705102 | T | -1.340115E-02 |
| rs6105852 | 20 | 17955153 | A | -1.010018E-02 |
| rs6046144 | 20 | 19477390 | G | 5.301662E-02 |
| rs78143514 | 20 | 19570596 | T | 1.616103E-02 |
| rs2076559 | 20 | 25187213 | A | 6.543287E-03 |
| rs736953 | 20 | 32415089 | T | 4.900223E-03 |
| rs4911414 | 20 | 32729444 | G | 6.426478E-03 |
| rs6029887 | 20 | 40573991 | G | -2.018575E-03 |
| rs2427104 | 20 | 60022160 | C | -1.262531E-03 |
| rs4925355 | 20 | 60773684 | T | 7.855580E-04 |
| rs911077 | 20 | 60963055 | C | 1.902862E-03 |
| rs2776068 | 21 | 18877791 | T | 4.342047E-03 |
| rs7277498 | 21 | 21030126 | G | 1.426382E-03 |
| rs232518 | 21 | 22746187 | C | 6.793664E-03 |
| rs2828483 | 21 | 25109093 | A | 3.867256E-03 |
| rs2195736 | 21 | 29125930 | G | -1.918025E-03 |
| rs8134439 | 21 | 33533540 | T | 2.406186E-03 |
| rs2834357 | 21 | 35407445 | C | -1.410619E-03 |
| rs13051441 | 21 | 39136297 | T | 1.749672E-05 |
| rs75603675 | 21 | 42879909 | A | -3.264542E-03 |
| rs220219 | 21 | 43444414 | G | -2.492435E-02 |
| rs10595 | 21 | 44324365 | A | 4.633490E-03 |
| rs9306108 | 21 | 46057391 | G | 9.377995E-03 |
| rs2838762 | 21 | 46487831 | C | -1.676603E-03 |
| rs2838821 | 21 | 46646546 | G | 3.293664E-03 |
| rs2236483 | 21 | 46926054 | C | -4.631841E-03 |
| rs701427 | 22 | 20233268 | C | 5.621065E-03 |
| rs9620328 | 22 | 24203799 | T | 8.990941E-04 |
| rs11090564 | 22 | 29704125 | T | -6.854720E-03 |
| rs11107 | 22 | 32875190 | A | -1.619096E-02 |
| rs11341975 | 22 | 32934713 | C | 5.333074E-03 |
| rs5749770 | 22 | 34642970 | G | -3.753860E-03 |
| rs6001368 | 22 | 39400832 | A | 2.867681E-03 |
| rs130649 | 22 | 39645316 | G | -1.206631E-03 |
| rs28605102 | 22 | 46329149 | T | -5.505853E-03 |
| rs8141744 | 22 | 46930107 | T | 5.308468E-03 |
| rs1467436 | 22 | 49335230 | T | 3.815355E-04 |
| rs2858649 | 22 | 49660492 | C | -5.326898E-04 |
| rs11703226 | 22 | 50658424 | C | 9.866306E-03 |
| rs470119 | 22 | 50966914 | C | -1.709419E-03 |
| rs359045 | 3 | 8434822 | G | 5.151200E-04 |
| rs61276202 | 3 | 14021080 | C | 1.354691E-03 |
| rs6794801 | 3 | 14531382 | A | -1.526039E-03 |
| rs1603022 | 3 | 22444521 | A | 1.371071E-03 |
| rs1077634 | 3 | 24305692 | G | -4.285408E-03 |
| rs3755652 | 3 | 27472936 | T | 1.533163E-03 |
| rs13075697 | 3 | 28712239 | A | -7.708159E-04 |
| rs1125186 | 3 | 28885804 | G | 4.446901E-04 |
| rs7650677 | 3 | 29203494 | G | 3.248800E-03 |
| rs987693 | 3 | 29305716 | G | 4.751462E-03 |
| rs7632357 | 3 | 32992203 | G | -1.403050E-03 |
| rs10490871 | 3 | 35667761 | G | 6.122161E-03 |
| rs73078787 | 3 | 41567009 | C | 2.620451E-03 |
| rs123509 | 3 | 42733468 | C | -9.739641E-03 |
| rs536119 | 3 | 43095101 | G | 1.515556E-02 |
| rs6779858 | 3 | 45148674 | G | -8.300009E-03 |
| rs17279437 | 3 | 45814094 | A | -1.443362E-02 |
| rs4434138 | 3 | 52556890 | G | -4.466513E-03 |
| rs678 | 3 | 52820981 | T | -2.176667E-02 |
| rs753819 | 3 | 58614393 | G | -1.196338E-03 |
| rs13075585 | 3 | 64062205 | C | 8.582701E-03 |
| rs6445523 | 3 | 65966296 | G | -7.270182E-04 |
| rs6549201 | 3 | 66600918 | T | -5.100138E-03 |
| rs6805929 | 3 | 66638886 | C | -3.912904E-03 |
| rs9877741 | 3 | 71195778 | A | -4.647891E-03 |
| rs9817445 | 3 | 71975432 | C | -6.674402E-03 |
| rs4076079 | 3 | 72209757 | C | 8.113687E-03 |
| rs10935564 | 3 | 98681275 | A | -1.653441E-04 |
| rs1131199 | 3 | 112059768 | G | 4.270179E-03 |
| rs11921691 | 3 | 113673125 | G | 2.544484E-03 |
| rs2903250 | 3 | 118649060 | T | 3.572613E-03 |
| rs1126828 | 3 | 128344786 | A | -3.823269E-03 |
| rs1869151 | 3 | 132614374 | A | 4.258835E-03 |
| rs2681689 | 3 | 141486583 | T | 3.731595E-03 |
| rs936194 | 3 | 142760690 | C | 7.178098E-03 |
| rs1876077 | 3 | 147359617 | C | 5.845598E-03 |
| rs6799981 | 3 | 150157729 | G | -6.187279E-03 |
| rs1354833 | 3 | 153051099 | G | -8.885082E-03 |
| rs9438 | 3 | 154018887 | C | -2.952461E-03 |
| rs11545169 | 3 | 184020542 | T | -3.729045E-03 |
| rs9839465 | 3 | 184326523 | T | -3.978435E-03 |
| rs1003995 | 3 | 187089031 | A | -4.724867E-03 |
| rs13059863 | 3 | 189065156 | C | -2.350175E-04 |
| rs11914582 | 3 | 189452211 | C | 1.155961E-03 |
| rs293833 | 3 | 191179193 | G | 6.474093E-03 |
| rs1563970 | 3 | 194349178 | T | -4.352089E-03 |
| rs1675923 | 3 | 194393208 | G | 1.441560E-03 |
| rs73205493 | 4 | 2460571 | T | -9.266896E-03 |
| rs35679952 | 4 | 2619709 | A | 3.151556E-03 |
| rs59953028 | 4 | 4256400 | A | -4.650205E-03 |
| rs2301788 | 4 | 6606864 | G | 8.134787E-03 |
| rs875373 | 4 | 13715903 | G | 3.421144E-03 |
| rs1807864 | 4 | 20151904 | C | -2.624450E-03 |
| rs7661530 | 4 | 21351260 | C | -1.172397E-03 |
| rs2199696 | 4 | 22661278 | T | -2.056544E-02 |
| rs317013 | 4 | 35404011 | T | 2.733459E-05 |
| rs7688524 | 4 | 36079976 | A | -4.449635E-04 |
| rs2925951 | 4 | 37910836 | A | 4.092419E-03 |
| rs2711941 | 4 | 39064162 | C | -2.052851E-03 |
| rs1480320 | 4 | 58706855 | C | 2.262478E-03 |
| rs1425660 | 4 | 68058793 | G | 3.191051E-03 |
| rs13133166 | 4 | 70600199 | G | 4.360856E-03 |
| rs4693809 | 4 | 81980966 | T | -5.428015E-03 |
| rs7677659 | 4 | 92431729 | T | -3.934498E-03 |
| rs7693022 | 4 | 95083575 | A | 5.525416E-03 |
| rs2866413 | 4 | 103557077 | A | -3.344834E-03 |
| rs4395588 | 4 | 108860978 | G | -5.509801E-03 |
| rs13102062 | 4 | 114064023 | C | 8.725888E-03 |
| rs3733526 | 4 | 120528327 | A | -9.321302E-03 |
| rs673857 | 4 | 132119814 | C | -6.712666E-03 |
| rs7672727 | 4 | 136842254 | C | 7.321151E-04 |
| rs6840566 | 4 | 138359842 | C | -3.366377E-03 |
| rs36097019 | 4 | 152201053 | A | 6.541826E-03 |
| rs3811834 | 4 | 153881835 | T | -6.546742E-03 |
| rs2305050 | 4 | 156273768 | C | 5.335942E-03 |
| rs11938430 | 4 | 163879822 | A | -3.616602E-04 |
| rs1170303 | 4 | 164620215 | A | -2.616309E-03 |
| rs13105490 | 4 | 164672357 | C | 5.169501E-03 |
| rs11724364 | 4 | 167043979 | T | -4.175480E-04 |
| rs6810619 | 4 | 173803914 | C | -4.630233E-03 |
| rs12502935 | 4 | 177113779 | C | -2.316005E-03 |
| rs6826896 | 4 | 188718859 | A | 7.535953E-03 |
| rs13149109 | 4 | 190094598 | G | -9.014784E-04 |
| rs13157694 | 5 | 2541190 | A | -7.528403E-04 |
| rs2935616 | 5 | 2726397 | C | -1.740811E-03 |
| rs1019747 | 5 | 5146377 | C | 3.243133E-03 |
| rs7714333 | 5 | 6317260 | G | 8.091172E-03 |
| rs7711377 | 5 | 7772199 | G | -1.073414E-02 |
| rs10434646 | 5 | 10987265 | T | 2.301946E-04 |
| rs4701678 | 5 | 17037781 | C | -6.143290E-03 |
| rs958502 | 5 | 37921235 | C | 1.032885E-03 |
| rs6863263 | 5 | 54254599 | A | 3.878942E-03 |
| rs30364 | 5 | 55829376 | T | 8.888646E-04 |
| rs3936510 | 5 | 55860866 | T | -4.794470E-03 |
| rs10939908 | 5 | 60879560 | C | 6.474239E-03 |
| rs3761966 | 5 | 70800475 | T | 4.070917E-03 |
| rs295691 | 5 | 73702310 | G | -1.153669E-04 |
| rs7710522 | 5 | 76968407 | A | -1.743342E-03 |
| rs532964 | 5 | 78340286 | G | -1.071469E-03 |
| rs55740951 | 5 | 88153874 | T | -1.138098E-02 |
| rs72781017 | 5 | 89593992 | G | -4.224860E-03 |
| rs12153602 | 5 | 97901912 | G | 8.524768E-04 |
| rs4703194 | 5 | 101202898 | C | 4.193239E-03 |
| rs26232 | 5 | 102596720 | T | -1.853452E-03 |
| rs17311608 | 5 | 102924875 | A | -5.055334E-03 |
| rs55716192 | 5 | 102979420 | C | -9.099808E-03 |
| rs149928825 | 5 | 112406785 | GCCTCGCGCTGTCTT | -2.042210E-03 |
| rs6865162 | 5 | 124047247 | G | 1.916472E-03 |
| rs251566 | 5 | 134800877 | A | 1.873693E-03 |
| rs436810 | 5 | 135781487 | T | 2.680664E-03 |
| rs4705135 | 5 | 146622157 | G | 2.923729E-03 |
| rs10043775 | 5 | 147805120 | C | -1.258633E-02 |
| rs11168048 | 5 | 147842353 | C | -7.241590E-03 |
| rs2900797 | 5 | 157402820 | T | -2.963088E-03 |
| rs4867902 | 5 | 168199624 | T | -1.768839E-03 |
| rs753860 | 5 | 168496090 | G | -5.250480E-04 |
| rs17676814 | 5 | 172791044 | T | 2.827527E-03 |
| rs2256596 | 6 | 7247248 | C | 2.435480E-03 |
| rs658222 | 6 | 10375970 | A | 2.529130E-03 |
| rs9382972 | 6 | 14501270 | G | 4.242362E-03 |
| rs16885 | 6 | 16306751 | A | 5.586542E-03 |
| rs3777712 | 6 | 17616556 | T | -3.933318E-03 |
| rs1042303 | 6 | 24437458 | C | -5.397436E-03 |
| rs72841509 | 6 | 26368279 | A | 8.718674E-03 |
| rs1011985 | 6 | 29385772 | C | -1.593871E-02 |
| rs1065076 | 6 | 31477681 | G | 9.060730E-04 |
| rs9267444 | 6 | 31483458 | A | 8.427508E-03 |
| rs2239526 | 6 | 31509432 | A | 5.627672E-03 |
| rs8084 | 6 | 32411035 | C | 2.764500E-03 |
| rs2495968 | 6 | 33926102 | T | 1.307035E-03 |
| rs13208903 | 6 | 36268998 | A | -3.808115E-03 |
| rs4713956 | 6 | 36275458 | C | 9.098390E-03 |
| rs13203701 | 6 | 37329800 | G | 7.541751E-03 |
| rs9471904 | 6 | 42671099 | T | -7.770807E-03 |
| rs10807452 | 6 | 52599135 | T | 3.355971E-04 |
| rs17678251 | 6 | 55708204 | A | 6.057941E-03 |
| rs3828767 | 6 | 70743251 | A | -3.349970E-03 |
| rs6915774 | 6 | 72456662 | C | 7.409009E-03 |
| rs6931045 | 6 | 72631747 | A | -5.581770E-03 |
| rs9342984 | 6 | 73598305 | T | 2.466886E-03 |
| rs9360611 | 6 | 73598337 | C | 1.228496E-02 |
| rs7760702 | 6 | 74842932 | A | 4.177711E-03 |
| rs239569 | 6 | 80725052 | C | -7.975379E-03 |
| rs13211072 | 6 | 84525784 | T | -1.787345E-03 |
| rs41288947 | 6 | 99894086 | G | 5.353371E-04 |
| rs66817403 | 6 | 99894360 | C | -2.592422E-04 |
| rs4896237 | 6 | 137447327 | C | 3.721401E-03 |
| rs6923492 | 6 | 146755324 | C | 8.471922E-04 |
| rs9397687 | 6 | 154462086 | T | -3.795346E-04 |
| rs9456340 | 6 | 159119811 | A | 1.454021E-02 |
| rs9365640 | 6 | 164450412 | A | 6.863985E-03 |
| rs2072767 | 6 | 167719436 | A | 8.311794E-03 |
| rs7764061 | 6 | 168694594 | T | 9.412384E-04 |
| rs6925614 | 6 | 168709385 | C | -3.749223E-03 |
| rs67899063 | 7 | 5171668 | T | 4.496069E-03 |
| rs7784370 | 7 | 7330622 | A | 7.356770E-04 |
| rs59497488 | 7 | 8742907 | G | 4.820723E-03 |
| rs7797070 | 7 | 9944175 | C | -1.935813E-03 |
| rs1557978 | 7 | 9965912 | C | -1.669902E-03 |
| rs34080 | 7 | 12963669 | G | -5.064925E-03 |
| rs6965130 | 7 | 22059834 | G | -6.058279E-04 |
| rs216703 | 7 | 28552665 | C | 1.258312E-02 |
| rs1610061 | 7 | 31631284 | G | 1.226286E-02 |
| rs7780515 | 7 | 32305274 | C | -1.219485E-03 |
| rs10243634 | 7 | 34792221 | G | 2.237703E-03 |
| rs1802074 | 7 | 37947103 | T | 2.959851E-03 |
| rs2215955 | 7 | 38469039 | A | 7.656774E-03 |
| rs62453608 | 7 | 39624269 | T | -2.443665E-03 |
| rs10282065 | 7 | 43649248 | A | 1.343447E-02 |
| rs6463400 | 7 | 47279128 | A | -5.810490E-03 |
| rs6978455 | 7 | 48451367 | C | 3.366078E-03 |
| rs73696925 | 7 | 48735819 | A | 5.955456E-03 |
| rs6965206 | 7 | 53216061 | C | -5.313956E-03 |
| rs67047829 | 7 | 64452738 | A | 1.261949E-02 |
| rs37608 | 7 | 73903036 | C | -8.778483E-04 |
| rs12538253 | 7 | 75388031 | C | -3.761524E-03 |
| rs7800072 | 7 | 84628989 | G | -9.625539E-03 |
| rs1468413 | 7 | 86433653 | A | 4.161205E-03 |
| rs803158 | 7 | 90741050 | G | -7.287931E-04 |
| rs2301680 | 7 | 93116299 | G | -1.411352E-03 |
| rs6465700 | 7 | 98342295 | A | -4.187182E-03 |
| rs71689664 | 7 | 99662511 | G | 1.468662E-02 |
| rs10225195 | 7 | 109167243 | C | 4.653326E-04 |
| rs34539765 | 7 | 114337877 | G | 7.623412E-04 |
| rs34912700 | 7 | 122897208 | T | 2.094028E-03 |
| rs712701 | 7 | 127251188 | G | 5.723719E-03 |
| rs4518582 | 7 | 136341233 | C | -1.688139E-03 |
| rs2948386 | 7 | 140125750 | C | -9.307148E-03 |
| rs1525734 | 7 | 144840057 | A | -7.983897E-03 |
| rs12703704 | 7 | 145249474 | G | 7.532649E-03 |
| rs1805123 | 7 | 150645534 | G | -3.134651E-03 |
| rs2303929 | 7 | 150761314 | A | 1.914002E-03 |
| rs6597489 | 7 | 155066000 | C | -7.351636E-03 |
| rs2364520 | 7 | 156042184 | C | -1.698034E-03 |
| rs11988412 | 8 | 3389347 | C | -2.707236E-04 |
| rs6558171 | 8 | 22487422 | C | -7.186835E-03 |
| rs11985023 | 8 | 22531463 | T | 3.078235E-03 |
| rs10107719 | 8 | 39190667 | T | 7.493673E-04 |
| rs35392772 | 8 | 57026229 | A | 1.145625E-02 |
| rs10957316 | 8 | 65446429 | C | 1.607881E-05 |
| rs7005485 | 8 | 78619137 | C | -1.279320E-02 |
| rs11984461 | 8 | 80980662 | A | 2.056853E-03 |
| rs10096021 | 8 | 83778775 | T | 7.653593E-03 |
| rs1496532 | 8 | 86253982 | T | 4.714934E-03 |
| rs13253402 | 8 | 102066725 | C | 9.225096E-03 |
| rs7002992 | 8 | 103676605 | C | -1.514164E-02 |
| rs2513902 | 8 | 103802649 | A | -2.867660E-03 |
| rs9785095 | 8 | 128064833 | G | 3.635690E-03 |
| rs13264712 | 8 | 131478440 | T | -2.554517E-03 |
| rs7820431 | 8 | 134571842 | A | -2.758014E-03 |
| rs62518386 | 8 | 134592408 | G | -7.524035E-04 |
| rs7835830 | 8 | 139165289 | C | -4.701147E-03 |
| rs307772 | 8 | 142146708 | C | 2.898046E-03 |
| rs7839934 | 8 | 144941181 | C | -2.547573E-04 |
| rs6475596 | 9 | 2201035 | A | -4.016065E-03 |
| rs7044370 | 9 | 2205924 | G | -9.843748E-04 |
| rs2025795 | 9 | 22347178 | A | 1.767565E-02 |
| rs1220065 | 9 | 25825005 | A | 9.821283E-04 |
| rs17081271 | 9 | 70999315 | C | -3.876035E-04 |
| rs9410809 | 9 | 82040514 | A | -4.372660E-03 |
| rs767563 | 9 | 83164953 | G | -1.237013E-04 |
| rs11139863 | 9 | 85511504 | G | -3.751307E-03 |
| rs2778942 | 9 | 87000938 | C | -6.686472E-03 |
| rs1387923 | 9 | 87640636 | G | -1.435706E-03 |
| rs4607675 | 9 | 96216409 | T | -5.822633E-03 |
| rs357564 | 9 | 98209594 | A | -8.692597E-03 |
| rs16916507 | 9 | 101258803 | A | -6.066250E-03 |
| rs4419863 | 9 | 103367481 | G | 1.412658E-03 |
| rs884975 | 9 | 109177722 | A | 9.932398E-03 |
| rs2269700 | 9 | 118163563 | T | -5.110271E-03 |
| rs7040792 | 9 | 119248993 | C | 3.887021E-03 |
| rs803915 | 9 | 119416731 | A | 1.639990E-02 |
| rs2273866 | 9 | 131702891 | G | -9.098138E-05 |
| rs3124511 | 9 | 131901458 | T | 4.330259E-04 |
| rs11243426 | 9 | 134434362 | T | 1.810678E-04 |
| rs10123855 | 9 | 134641344 | T | 9.097840E-04 |
| rs944807 | 9 | 135397773 | C | 1.450097E-02 |
| rs4962095 | 9 | 136031751 | T | 4.030494E-03 |
| rs8176719 | 9 | 136132908 | TC | 3.599917E-03 |
| rs7037251 | 9 | 138391299 | A | -1.293314E-03 |
| rs28712661 | 9 | 139230067 | G | -7.956011E-03 |
| rs7037849 | 9 | 139973820 | T | -1.575261E-03 |

RSID: variant identifier; CHR: chromosome; BP: base-pair position; Effect: effect allele used for scoring; Weight: per-SNP weight in the PRS; PRS: polygenic risk score; DR: diabetic retinopathy.

Table S3. Summary of reported susceptibility loci used for the construction of the weighted PRS for DR.

| RSID | CHR | BP | Effect | Weight |
| --- | --- | --- | --- | --- |
| rs11711732 | 3 | 131382778 | T | 3.407915E-04 |
| rs11916885 | 3 | 186914144 | A | 4.304244E-03 |
| rs6448475 | 4 | 26831827 | T | -7.131335E-04 |
| rs16896723 | 5 | 66701863 | A | 3.348692E-03 |
| rs17109807 | 5 | 148600845 | T | 4.532326E-03 |
| rs201018 | 6 | 6666844 | C | 4.391878E-03 |
| rs2596473 | 6 | 31430799 | T | -2.890713E-04 |
| rs2855812 | 6 | 31472720 | T | 3.607379E-02 |
| rs2857602 | 6 | 31533378 | A | 1.245447E-03 |
| rs2736173 | 6 | 31589735 | T | 1.460575E-02 |
| rs2736157 | 6 | 31600820 | G | 8.328016E-04 |
| rs3117583 | 6 | 31619576 | G | 4.019680E-03 |
| rs652888 | 6 | 31851234 | G | 1.272218E-02 |
| rs1150755 | 6 | 32038550 | T | 1.632588E-02 |
| rs915894 | 6 | 32190390 | G | 2.429392E-02 |
| rs9501398 | 6 | 32202597 | T | 4.560912E-03 |
| rs3129871 | 6 | 32406342 | C | 9.930176E-05 |
| rs3129716 | 6 | 32657436 | C | 7.496073E-02 |
| rs3957146 | 6 | 32681530 | C | 4.266301E-01 |
| rs3998159 | 6 | 32682019 | C | 2.368265E-02 |
| rs9275614 | 6 | 32684257 | G | 1.220157E-02 |
| rs11969718 | 6 | 156286386 | A | 2.292008E-03 |
| rs12698637 | 7 | 67420743 | G | -4.253788E-03 |
| rs4730074 | 7 | 104892139 | C | -4.551940E-03 |
| rs17289756 | 7 | 105712320 | A | 2.121555E-03 |
| rs17066956 | 8 | 3555657 | T | 5.454997E-03 |
| rs2205262 | 8 | 117011884 | A | 6.640552E-04 |
| rs10811661 | 9 | 22134094 | C | -4.011745E-02 |
| rs12684650 | 9 | 139110654 | T | 9.343184E-04 |
| rs12243326 | 10 | 114788815 | C | 2.142957E-02 |
| rs12255372 | 10 | 114808902 | T | 8.832708E-03 |
| rs3842752 | 11 | 2181073 | A | -2.186836E-04 |
| rs16917237 | 11 | 27702383 | T | -8.388265E-04 |
| rs12422853 | 12 | 105063882 | A | 2.816880E-04 |
| rs10132309 | 14 | 101464984 | T | -1.184163E-05 |
| rs7501939 | 17 | 36101156 | C | -2.108794E-02 |
| rs1788234 | 18 | 67566587 | T | 1.726682E-03 |
| rs6046214 | 20 | 19613399 | G | -1.755440E-03 |
| rs138628 | 22 | 44988209 | C | -7.945212E-04 |

RSID: variant identifier; CHR: chromosome; BP: base-pair position; Effect: effect allele used for scoring; Weight: per-SNP weight in the PRS; PRS: polygenic risk score; DR: diabetic retinopathy.

Table S4. Summary of reported susceptibility loci used for the construction of the weighted PRS for glaucoma.

| RSID | CHR | BP | Effect | Weight |
| --- | --- | --- | --- | --- |
| rs10737907 | 1 | 15509852 | A | 7.919444E-02 |
| rs75240370 | 1 | 38304192 | A | -6.242159E-02 |
| rs4634868 | 1 | 38465315 | G | 4.357006E-02 |
| rs59571065 | 1 | 38518043 | A | -5.597375E-02 |
| rs12728919 | 1 | 45976249 | G | -5.762796E-02 |
| rs41313369 | 1 | 53980389 | C | -5.850601E-02 |
| rs11590557 | 1 | 54789772 | G | 1.023758E-01 |
| rs10736397 | 1 | 62109420 | A | -4.523693E-02 |
| rs72935320 | 1 | 80931508 | C | 1.755099E-01 |
| rs61800659 | 1 | 86803735 | T | -7.935654E-02 |
| rs112901772 | 1 | 88211845 | C | -1.058968E-01 |
| rs74496297 | 1 | 92281833 | C | 1.019212E-01 |
| rs67043929 | 1 | 94040287 | T | 5.935629E-02 |
| rs78099894 | 1 | 117397250 | A | -8.517139E-02 |
| rs11205303 | 1 | 149906413 | T | 4.426995E-02 |
| rs11589479 | 1 | 155033308 | G | 6.658114E-02 |
| rs6674639 | 1 | 165758106 | A | 6.480434E-02 |
| rs6425434 | 1 | 171785238 | G | 1.302790E-01 |
| rs7520165 | 1 | 172177789 | T | -1.542964E-01 |
| rs61829344 | 1 | 197683734 | A | 5.967212E-02 |
| rs2737659 | 1 | 200053219 | C | -3.958408E-02 |
| rs875093 | 1 | 204600719 | A | 4.009434E-02 |
| rs2789731 | 1 | 216923473 | C | -4.536445E-02 |
| rs7079 | 1 | 230838331 | G | -4.200216E-02 |
| rs2160204 | 2 | 10515219 | C | -9.000416E-02 |
| rs7581217 | 2 | 25524944 | T | -3.658341E-02 |
| rs6741499 | 2 | 28369436 | C | -8.260851E-02 |
| rs11904654 | 2 | 33334699 | T | -5.436442E-02 |
| rs77234141 | 2 | 43390840 | T | -1.051148E-01 |
| rs17278051 | 2 | 55749492 | C | -1.041980E-01 |
| rs6709302 | 2 | 60727629 | G | -3.735752E-02 |
| rs4671599 | 2 | 64829465 | A | -3.773656E-02 |
| rs62149622 | 2 | 73535292 | A | -4.178652E-02 |
| rs298917 | 2 | 98709684 | T | 4.426203E-02 |
| rs13403558 | 2 | 110885620 | A | 5.171102E-02 |
| rs9308627 | 2 | 112397505 | T | 4.495475E-02 |
| rs75239367 | 2 | 153282016 | T | 1.245543E-01 |
| rs72924348 | 2 | 192483003 | G | 6.233592E-02 |
| rs62188040 | 2 | 213723345 | C | 1.011931E-01 |
| rs114315112 | 2 | 233400171 | A | 1.191506E-01 |
| rs13010612 | 2 | 233554720 | G | -1.055316E-01 |
| rs72982602 | 2 | 239460677 | C | -1.097920E-01 |
| rs4247179 | 2 | 242585616 | G | 3.749069E-02 |
| rs71313303 | 3 | 25549012 | A | -8.780212E-02 |
| rs75877123 | 3 | 29413352 | A | -5.613156E-02 |
| rs2371996 | 3 | 30200658 | A | 3.818948E-02 |
| rs9681018 | 3 | 33529515 | C | 5.064221E-02 |
| rs77190659 | 3 | 42246948 | A | -9.660621E-02 |
| rs34673873 | 3 | 46264059 | C | -5.773848E-02 |
| rs73082903 | 3 | 50272712 | G | 7.470395E-02 |
| rs1574016 | 3 | 61546765 | T | -4.054113E-02 |
| rs77596649 | 3 | 62602190 | T | 1.125037E-01 |
| rs80019555 | 3 | 67196001 | T | -8.321070E-02 |
| rs74806059 | 3 | 77419337 | C | 1.304084E-01 |
| rs77022800 | 3 | 86355821 | G | 1.154310E-01 |
| rs114105957 | 3 | 88382904 | T | 1.006904E-01 |
| rs76325960 | 3 | 115551968 | G | 1.274189E-01 |
| rs956929 | 3 | 122761183 | T | -9.235366E-02 |
| rs115873098 | 3 | 134589411 | G | -1.472246E-01 |
| rs11710845 | 3 | 150065280 | C | 7.209834E-02 |
| rs9827448 | 3 | 159351238 | C | 7.576612E-02 |
| rs61738871 | 3 | 169539812 | C | -5.877631E-02 |
| rs17521080 | 3 | 171991746 | T | -6.656923E-02 |
| rs62290721 | 3 | 186166074 | A | 1.280309E-01 |
| rs116783230 | 3 | 186167839 | C | 1.388775E-01 |
| rs115263492 | 3 | 186172959 | C | -1.397014E-01 |
| rs12495941 | 3 | 186568180 | G | 4.568572E-02 |
| rs59285533 | 3 | 188202323 | C | -1.208594E-01 |
| rs114740145 | 4 | 7802418 | C | -9.300830E-02 |
| rs7440527 | 4 | 7826824 | T | -5.522487E-02 |
| rs28689523 | 4 | 7844081 | T | 6.503304E-02 |
| rs1618403 | 4 | 8216391 | C | 3.588086E-02 |
| rs78287220 | 4 | 53890473 | A | 1.438053E-01 |
| rs11932595 | 4 | 56323597 | A | -3.875796E-02 |
| rs112920510 | 4 | 95488920 | G | -9.842762E-02 |
| rs72660629 | 4 | 105322746 | C | 4.873557E-02 |
| rs13126426 | 4 | 111960374 | T | 1.277952E-01 |
| rs78284654 | 4 | 115301900 | T | -1.375978E-01 |
| rs116103199 | 4 | 126246434 | G | 1.040842E-01 |
| rs8192065 | 4 | 140600761 | T | 1.813439E-01 |
| rs34901370 | 4 | 146234605 | T | 4.448617E-02 |
| rs75902705 | 4 | 176859108 | C | 1.766578E-01 |
| rs72691513 | 4 | 184470852 | C | 9.624375E-02 |
| rs114234330 | 5 | 1732732 | G | -1.341126E-01 |
| rs10513023 | 5 | 9569067 | C | 1.887515E-01 |
| rs10461928 | 5 | 33944217 | G | 3.842462E-02 |
| rs35117676 | 5 | 38904082 | T | -1.735679E-01 |
| rs10472028 | 5 | 57254667 | A | 5.979474E-02 |
| rs9790869 | 5 | 64858580 | G | -4.465401E-02 |
| rs3805452 | 5 | 71495041 | T | 4.282095E-02 |
| rs77881520 | 5 | 75398520 | A | -1.786506E-01 |
| rs7712710 | 5 | 99003698 | T | -7.053320E-02 |
| rs41295699 | 5 | 137907873 | A | -8.276169E-02 |
| rs2961692 | 5 | 141564716 | A | -4.818418E-02 |
| rs34221241 | 5 | 180057293 | T | -5.865306E-02 |
| rs722585 | 6 | 1775863 | G | 7.931475E-02 |
| rs77026064 | 6 | 1837419 | T | 1.890216E-01 |
| rs76152304 | 6 | 16708459 | G | -6.229991E-02 |
| rs2535336 | 6 | 30810654 | A | -4.966563E-02 |
| rs885701 | 6 | 31143900 | A | -1.883218E-01 |
| rs9267954 | 6 | 32213052 | A | -4.621860E-02 |
| rs77057879 | 6 | 34453835 | T | -1.193620E-01 |
| rs2179759 | 6 | 46145899 | C | 5.423449E-02 |
| rs2206277 | 6 | 50798526 | C | -8.049308E-02 |
| rs9474687 | 6 | 53795480 | T | 5.319639E-02 |
| rs642635 | 6 | 70942660 | T | -5.399839E-02 |
| rs76122922 | 6 | 78242765 | G | -1.228513E-01 |
| rs10455150 | 6 | 85834607 | G | 4.002186E-02 |
| rs34902403 | 6 | 116073847 | A | 9.536137E-02 |
| rs118175036 | 6 | 125805583 | C | 1.028677E-01 |
| rs111750937 | 6 | 150991166 | G | -9.417310E-02 |
| rs118128369 | 6 | 161916039 | T | 1.383083E-01 |
| rs117600863 | 6 | 170520151 | T | -8.646245E-02 |
| rs10260511 | 7 | 14237240 | C | -6.636258E-02 |
| rs73075505 | 7 | 26713506 | C | 1.213133E-01 |
| rs73132704 | 7 | 39870019 | A | -1.191748E-01 |
| rs258076 | 7 | 40944385 | A | -3.642932E-02 |
| rs17690388 | 7 | 92345357 | G | -8.961496E-02 |
| rs4729100 | 7 | 93529615 | C | 3.633921E-02 |
| rs117501810 | 7 | 101153000 | A | -9.957518E-02 |
| rs2041475 | 7 | 103188061 | C | 5.285760E-02 |
| rs116494445 | 7 | 103356820 | T | -1.007569E-01 |
| rs149054673 | 7 | 116212245 | G | -2.177554E-01 |
| rs74915662 | 7 | 119583865 | C | -1.018412E-01 |
| rs17536693 | 7 | 120723436 | G | 1.441997E-01 |
| rs73158836 | 7 | 132132201 | C | -7.854882E-02 |
| rs62465255 | 7 | 132363387 | G | -5.887576E-02 |
| rs76020419 | 8 | 6360289 | G | 1.299626E-01 |
| rs13257716 | 8 | 13396357 | C | -1.116360E-01 |
| rs11995050 | 8 | 25310042 | A | 4.476892E-02 |
| rs79983864 | 8 | 30764154 | G | -9.585364E-02 |
| rs10504247 | 8 | 58837634 | A | -6.580665E-02 |
| rs11776708 | 8 | 67638069 | A | -6.066249E-02 |
| rs1912391 | 8 | 78550000 | T | 5.943493E-02 |
| rs62517716 | 8 | 79413123 | C | 5.985812E-02 |
| rs59214414 | 8 | 79763522 | G | 1.250601E-01 |
| rs72674666 | 8 | 100594204 | C | -7.243969E-02 |
| rs76878476 | 8 | 104572885 | G | -1.117125E-01 |
| rs35456088 | 8 | 108012535 | G | 1.008952E-01 |
| rs76398386 | 8 | 108367678 | C | -1.032382E-01 |
| rs10105614 | 8 | 108532794 | A | -7.822748E-02 |
| rs62520133 | 8 | 114582092 | C | 1.137869E-01 |
| rs2001966 | 8 | 124829395 | T | -4.700980E-02 |
| rs117850821 | 8 | 139214840 | G | 1.307128E-01 |
| rs112648016 | 9 | 4552102 | G | -8.824438E-02 |
| rs74903566 | 9 | 21941315 | G | 9.665322E-02 |
| rs10965250 | 9 | 22133284 | G | 4.915070E-02 |
| rs72735243 | 9 | 34501938 | C | 4.352130E-02 |
| rs67071328 | 9 | 107717705 | C | 7.632871E-02 |
| rs2263594 | 9 | 108354401 | G | -8.229077E-02 |
| rs2025875 | 9 | 112596911 | G | -4.404043E-02 |
| rs61751937 | 9 | 113312231 | G | -1.544487E-01 |
| rs2567706 | 9 | 116930194 | A | -4.841189E-02 |
| rs78043174 | 9 | 119285892 | G | -1.385120E-01 |
| rs2416864 | 9 | 125254013 | A | 4.654893E-02 |
| rs11793585 | 9 | 129260088 | C | 1.155650E-01 |
| rs12783492 | 10 | 5563929 | G | -4.030372E-02 |
| rs11007653 | 10 | 29850081 | A | -3.712757E-02 |
| rs2795590 | 10 | 30351203 | A | 1.160979E-01 |
| rs796076 | 10 | 31908498 | G | 3.770476E-02 |
| rs34764480 | 10 | 44439740 | C | -9.403007E-02 |
| rs753247 | 10 | 63342433 | C | 6.952797E-02 |
| rs12246748 | 10 | 65392260 | C | -3.923714E-02 |
| rs76544144 | 10 | 77658651 | T | -1.128414E-01 |
| rs61861449 | 10 | 94369982 | C | -1.396600E-01 |
| rs2274224 | 10 | 96039597 | G | -7.737820E-02 |
| rs4751674 | 10 | 116139029 | T | -4.737963E-02 |
| rs80244099 | 10 | 118416080 | G | -7.092923E-02 |
| rs79312974 | 11 | 28071201 | C | -1.557880E-01 |
| rs79167034 | 11 | 46814844 | T | -1.181457E-01 |
| rs60147425 | 11 | 47806206 | G | -2.385401E-01 |
| rs117353933 | 11 | 50344221 | G | -8.129459E-02 |
| rs11607910 | 11 | 56334457 | G | -5.088400E-02 |
| rs754532 | 11 | 65747057 | G | -4.834779E-02 |
| rs2226615 | 11 | 75956348 | G | 4.224692E-02 |
| rs17517124 | 11 | 114100609 | T | -7.805534E-02 |
| rs3819531 | 12 | 2436801 | C | 4.708381E-02 |
| rs5442 | 12 | 6954864 | G | -8.913854E-02 |
| rs71448619 | 12 | 22179668 | G | 1.363763E-01 |
| rs11048174 | 12 | 25835245 | C | 8.488026E-02 |
| rs78820474 | 12 | 27522240 | G | -1.000352E-01 |
| rs78404378 | 12 | 54121585 | A | 9.159445E-02 |
| rs11106868 | 12 | 93523665 | G | 6.957117E-02 |
| rs2196425 | 12 | 94112078 | C | 5.068714E-02 |
| rs76465489 | 13 | 22813108 | T | 5.332259E-02 |
| rs117878987 | 13 | 27316298 | A | -1.520923E-01 |
| rs1924810 | 13 | 44964182 | T | -4.562978E-02 |
| rs9530143 | 13 | 73639371 | G | 6.427936E-02 |
| rs118140727 | 13 | 76471794 | G | -1.740620E-01 |
| rs2383436 | 14 | 33539762 | A | 3.701562E-02 |
| rs8016787 | 14 | 33584752 | G | 4.195061E-02 |
| rs78269823 | 14 | 39264849 | C | -6.618804E-02 |
| rs61755579 | 14 | 50655307 | C | 1.691850E-01 |
| rs17180111 | 14 | 65202852 | G | 8.888739E-02 |
| rs3784095 | 14 | 68723484 | C | -4.256538E-02 |
| rs79050575 | 14 | 76227652 | A | -1.053905E-01 |
| rs75560721 | 14 | 84257037 | G | 1.327555E-01 |
| rs1805078 | 14 | 88450770 | G | -7.728834E-02 |
| rs78798291 | 14 | 91430034 | A | 9.728361E-02 |
| rs4905331 | 14 | 95947747 | G | 7.117754E-02 |
| rs4587923 | 15 | 68762297 | C | -4.433500E-02 |
| rs627680 | 15 | 71410495 | A | 3.512885E-02 |
| rs113232537 | 15 | 71585576 | A | -1.539315E-01 |
| rs115094589 | 15 | 74197029 | G | 1.464173E-01 |
| rs938682 | 15 | 78896547 | G | -5.016968E-02 |
| rs2280468 | 15 | 89381556 | C | 3.786121E-02 |
| rs28600760 | 15 | 92119411 | T | -8.923996E-02 |
| rs117799253 | 15 | 94207857 | C | -1.180760E-01 |
| rs10520789 | 15 | 96141867 | G | 5.639128E-02 |
| rs28612945 | 15 | 99458902 | C | 4.793314E-02 |
| rs62031360 | 16 | 9246545 | C | 9.007993E-02 |
| rs117671848 | 16 | 18791970 | G | -1.255721E-01 |
| rs61249331 | 16 | 49032921 | C | 1.396990E-01 |
| rs16947792 | 16 | 49687824 | A | 4.960028E-02 |
| rs9934971 | 16 | 51326478 | C | -4.910586E-02 |
| rs78818722 | 16 | 67991972 | C | -7.512007E-02 |
| rs1597296 | 16 | 77695251 | G | -4.956344E-02 |
| rs2279349 | 16 | 89350178 | G | 6.378509E-02 |
| rs7210608 | 17 | 9874244 | A | 5.356323E-02 |
| rs72812844 | 17 | 10153190 | G | -1.401222E-01 |
| rs2074877 | 17 | 10223714 | T | -4.115213E-02 |
| rs72813627 | 17 | 29530876 | G | -6.695080E-02 |
| rs10432043 | 17 | 44869567 | C | 3.871609E-02 |
| rs118086135 | 17 | 44891899 | G | -1.115679E-01 |
| rs118036504 | 17 | 46073854 | T | -1.467481E-01 |
| rs75535096 | 17 | 47340957 | C | -1.161634E-01 |
| rs56992242 | 17 | 59664673 | G | -4.438738E-02 |
| rs12953146 | 17 | 60905859 | A | 5.687727E-02 |
| rs12963050 | 18 | 807975 | T | -4.319165E-02 |
| rs79850096 | 18 | 4739271 | A | 9.745261E-02 |
| rs10853528 | 18 | 42526328 | C | 4.082035E-02 |
| rs1047922 | 18 | 74070562 | T | -6.842397E-02 |
| rs12981904 | 19 | 13248856 | T | 4.751208E-02 |
| rs2305753 | 19 | 17180033 | C | 5.150049E-02 |
| rs73537455 | 19 | 19299431 | C | 6.492787E-02 |
| rs11667974 | 19 | 52218958 | C | 5.821355E-02 |
| rs512452 | 20 | 60944084 | A | 5.246841E-02 |
| rs77652771 | 21 | 32688974 | C | -1.561261E-01 |
| rs77845718 | 22 | 27895208 | G | 1.482862E-01 |
| rs738722 | 22 | 29130012 | T | -9.125233E-02 |
| rs117079136 | 22 | 41757770 | C | -8.570985E-02 |
| rs76645083 | 22 | 45675375 | C | 8.247757E-02 |
| rs13268 | 22 | 45996298 | A | 1.309767E-01 |

RSID: variant identifier; CHR: chromosome; BP: base-pair position; Effect: effect allele used for scoring; Weight: per-SNP weight in the PRS; PRS: polygenic risk score.

Table S5. Coding and definition information of variable in the UK Biobank.

| Variables | Definition | Field ID in UK Biobank |
| --- | --- | --- |
| AMD | No; Yes | ICD10-H35.3 (41270); ICD9-3625 (41271) |
| Cataract | No; Yes | ICD10-H25.0, H25.1, H25.2, H25.8, H25.9, H26.0, H26.2, H26.3, H26.4, H26.8, H26.9, H28.0, H28.1, H28.2 (41270); ICD9-3660, 3662, 3663, 3664, 3665, 3668, 3669 (41271) |
| DR | No; Yes | ICD10-H36.0 (41270); ICD9-3620 (41271) |
| Glaucoma | No; Yes | ICD10-H40.0, H40.1, H40.2, H40.8, H40.9 (41270); ICD9-3650, 3651, 3652, 3658, 3659 (41271) |
| Chronological age | Age (years) | Age at recruitment (21022) |
| Sex | Female; Male | Sex (31) |
| Ethnicity | White; Mixed; Asian; Black; Others | Ethnic background (21000) |
| Education level | Degree level or professional education; Other levels | Qualifications (6138) |
| BMI | Body mass index (kg/m^2^) | Body mass index (21001) |
| TDI | Townsend deprivation index | Townsend deprivation index at recruitment (22189) |
| Physical activity | Low; Moderate; High | Summed MET minutes per week for all activity (22040) |
| Sleep duration | Short; Moderate; Long | Sleep duration (1160) |
| Healthy diet | No; Yes | Fresh fruit intake (1309); Dried fruit intake (1319); Cooked vegetable intake (1289); Salad / raw vegetable intake (1299); Bread intake (1438); Bread type (1448); Cereal intake (1458); Cereal type (1468); Oily fish intake (1329); Non-oily fish intake (1339); Cheese intake (1408); Milk type used (1418); Spread type (1428); Non-butter spread type details (2654); Processed meat intake (1439); Age when last ate meat (3680); Poultry intake (1359); Beef intake (1369); Lamb/mutton intake (1379); Pork intake (1389); Never eat eggs, dairy, wheat, sugar (6144) |
| Smoking | No; Yes | Current tobacco smoking (1239); Past tobacco smoking (1249); Light smokers, at least 100 smokes in lifetime (2644) |
| Alcohol | No; Yes | Alcohol intake frequency (1558); Average weekly red wine intake (1568); Average weekly champagne plus white wine intake (1578); Average weekly beer plus cider intake (1588); Average weekly spirits intake (1598); Average weekly fortified wine intake (1608); Average weekly intake of other alcoholic drinks (5364) |
| Citrate | Citrate (µmol/L) | Citrate (23473) |
| Isoleucine | Isoleucine (µmol/L) | Isoleucine (23465) |
| Glycoprotein acetyls | Glycoprotein acetyls (µmol/L) | Glycoprotein acetyls (23480) |
| Leucine | Leucine (µmol/L) | Leucine (23466) |
| Small HDL particle number | Concentration of Small HDL Particles (µmol/L) | Concentration of Small HDL Particles (23572) |
| Valine | Valine (µmol/L) | Valine (23467) |

ICD: International Classification of Diseases; AMD: age-related macular degeneration; DR: diabetic retinopathy.

Table S6. Association between PRS and AMD risk.

| Exposures | Model 1 | | Model 2 | |
| --- | --- | --- | --- | --- |
|  | HR (95% CI) | P-value | HR (95% CI) | P-value |
| PRS-CS (continuous) | | | | |
| Per 1 SD increase | 1.36 (1.32–1.40) | <0.001 | 1.36 (1.32–1.40) | <0.001 |
| PRS-CS (category) | | | | |
| Low risk | 1.00 (Reference) |  | 1.00 (Reference) |  |
| Intermediate risk | 1.27 (1.16–1.38) | <0.001 | 1.26 (1.16–1.38) | <0.001 |
| High genetic risk | 2.16 (1.96–2.38) | <0.001 | 2.16 (1.96–2.38) | <0.001 |
| Weighted PRS (continuous) | | | | |
| Per 1 SD increase | 1.36 (1.32–1.40) | <0.001 | 1.36 (1.32–1.40) | <0.001 |
| Weighted PRS (category) | | | | |
| Low risk | 1.00 (Reference) |  | 1.00 (Reference) |  |
| Intermediate risk | 1.19 (1.09–1.30) | <0.001 | 1.19 (1.09–1.30) | <0.001 |
| High genetic risk | 2.07 (1.88–2.28) | <0.001 | 2.07 (1.88–2.28) | <0.001 |

Model 1 adjusted for chronological age, sex, ethnicity, education, BMI, and Townsend deprivation index. Model 2 Further adjusted for physical activity, sleep duration, smoking, alcohol consumption, and healthy diet. PRS: polygenic risk score; CS: continuous shrinkage; BMI: body mass index; AMD: age-related macular degeneration.

Table S7. Association between PRS and cataract risk.

| Exposures | Model 1 | | Model 2 | |
| --- | --- | --- | --- | --- |
|  | HR (95% CI) | P-value | HR (95% CI) | P-value |
| PRS-CS (continuous) | | | | |
| Per 1 SD increase | 1.01 (0.99–1.02) | 0.397 | 1.01 (0.99–1.02) | 0.406 |
| PRS-CS (category) | | | | |
| Low risk | 1.00 (Reference) |  | 1.00 (Reference) |  |
| Intermediate risk | 1.01 (0.96–1.05) | 0.773 | 1.01 (0.96–1.05) | 0.778 |
| High genetic risk | 1.00 (0.95–1.06) | 0.872 | 1.00 (0.95–1.06) | 0.872 |
| Weighted PRS (continuous) | | | | |
| Per 1 SD increase | 1.16 (1.14–1.18) | <0.001 | 1.16 (1.14–1.18) | <0.001 |
| Weighted PRS (category) | | | | |
| Low risk | 1.00 (Reference) |  | 1.00 (Reference) |  |
| Intermediate risk | 1.24 (1.18–1.29) | <0.001 | 1.24 (1.18–1.29) | <0.001 |
| High genetic risk | 1.52 (1.44–1.61) | <0.001 | 1.52 (1.44–1.61) | <0.001 |

Model 1 adjusted for chronological age, sex, ethnicity, education, BMI, and Townsend deprivation index. Model 2 Further adjusted for physical activity, sleep duration, smoking, alcohol consumption, and healthy diet; PRS: polygenic risk score; CS: continuous shrinkage. BMI: body mass index.

Table S8. Association between PRS and DR risk.

| Exposures | Model 1 | | Model 2 | |
| --- | --- | --- | --- | --- |
|  | HR (95% CI) | P-value | HR (95% CI) | P-value |
| PRS-CS (continuous) | | | | |
| Per 1 SD increase | 1.56 (1.48–1.64) | <0.001 | 1.55 (1.47–1.63) | <0.001 |
| PRS-CS (category) | | | | |
| Low risk | 1.00 (Reference) |  | 1.00 (Reference) |  |
| Intermediate risk | 1.90 (1.58–2.29) | <0.001 | 1.89 (1.57–2.27) | <0.001 |
| High genetic risk | 3.57 (2.95–4.33) | <0.001 | 3.51 (2.90–4.25) | <0.001 |
| Weighted PRS (continuous) | | | | |
| Per 1 SD increase | 1.17 (1.12–1.23) | <0.001 | 1.17 (1.12–1.23) | <0.001 |
| Weighted PRS (category) | | | | |
| Low risk | 1.00 (Reference) |  | 1.00 (Reference) |  |
| Intermediate risk | 1.40 (1.20–1.62) | <0.001 | 1.40 (1.20–1.62) | <0.001 |
| High genetic risk | 1.68 (1.42–1.99) | <0.001 | 1.67 (1.41–1.98) | <0.001 |

Model 1 adjusted for chronological age, sex, ethnicity, education, BMI, and Townsend deprivation index. Model 2 Further adjusted for physical activity, sleep duration, smoking, alcohol consumption, and healthy diet. PRS: polygenic risk score; CS: continuous shrinkage; BMI: body mass index; DR: diabetic retinopathy.

Table S9. Association between PRS and glaucoma risk.

| Exposures | Model 1 | | Model 2 | |
| --- | --- | --- | --- | --- |
|  | HR (95% CI) | P-value | HR (95% CI) | P-value |
| PRS-CS (continuous) | | | | |
| Per 1 SD increase | 1.37 (1.34–1.41) | <0.001 | 1.37 (1.34–1.41) | <0.001 |
| PRS-CS (category) | | | | |
| Low risk | 1.00 (Reference) |  | 1.00 (Reference) |  |
| Intermediate risk | 1.53 (1.41–1.66) | <0.001 | 1.53 (1.41–1.66) | <0.001 |
| High genetic risk | 2.36 (2.16–2.58) | <0.001 | 2.36 (2.16–2.58) | <0.001 |
| Weighted PRS (continuous) | | | | |
| Per 1 SD increase | 1.27 (1.24–1.31) | <0.001 | 1.27 (1.24–1.31) | <0.001 |
| Weighted PRS (category) | | | | |
| Low risk | 1.00 (Reference) |  | 1.00 (Reference) |  |
| Intermediate risk | 1.46 (1.35–1.58) | <0.001 | 1.46 (1.35–1.58) | <0.001 |
| High genetic risk | 2.00 (1.83–2.19) | <0.001 | 2.00 (1.83–2.19) | <0.001 |

Model 1 adjusted for chronological age, sex, ethnicity, education, BMI, and Townsend deprivation index. Model 2 Further adjusted for physical activity, sleep duration, smoking, alcohol consumption, and healthy diet. PRS: polygenic risk score; CS: continuous shrinkage; BMI: body mass index.

Table S10. Additive interaction between MVX and genetic risk in incident age-related eye diseases.

| Category | Additive interaction | | | |
| --- | --- | --- | --- | --- |
|  | Intermediate PRS | | High PRS | |
|  | RERI (95%CI) | AP (95%CI) | RERI (95%CI) | AP (95%CI) |
| AMD | | | | |
| High MVX | 0.11 (-0.08–0.28) | 0.08 (-0.06–0.22) | 0.42 (0.15–0.70) | 0.18 (0.07–0.30) |
| Cataract | | | | |
| High MVX | 0.05 (-0.06–0.14) | 0.04 (-0.04–0.11) | 0.10 (-0.03–0.23) | 0.06 (-0.02–0.14) |
| DR | | | | |
| High MVX | 0.03 (-0.45–0.44) | 0.02 (-0.21–0.24) | 0.39 (-0.37–1.13) | 0.10 (-0.09–0.28) |
| Glaucoma | | | | |
| High MVX | 0.01 (-0.20–0.18) | 0 (-0.12–0.12) | -0.21 (-0.51–0.06) | -0.09 (-0.22–0.02) |

Values are the RERI and the AP with 95% CIs for MVX higher vs. lower within strata of PRS. Chronological age, sex, ethnicity, education, BMI, and Townsend deprivation index, physical activity, sleep duration, smoking, alcohol consumption, and healthy diet were adjusted in the analyses. PRS: polygenic risk score; AMD: age-related macular degeneration; DR: diabetic retinopathy; RERI: relative excess risk due to interaction; AP: attributable proportion.
